# Roles of the lamin A-specific tail region in the localization to sites of nuclear envelope rupture

**DOI:** 10.1093/pnasnexus/pgae527

**Published:** 2024-11-21

**Authors:** Yohei Kono, Chan-Gi Pack, Takehiko Ichikawa, Arata Komatsubara, Stephen A Adam, Keisuke Miyazawa, Loïc Rolas, Sussan Nourshargh, Ohad Medalia, Robert D Goldman, Takeshi Fukuma, Hiroshi Kimura, Takeshi Shimi

**Affiliations:** Cell Biology Center, Institute of Innovative Research, Tokyo Institute of Technology, Yokohama 226-8503, Japan; Nano Life Science Institute (WPI-NanoLSI), Kanazawa University, Kanazawa 920-1192, Japan; Faculty of Frontier Engineering, Institute of Science and Engineering, Kanazawa University, Kanazawa 920-1192, Japan; Convergence Medicine Research Center, Asan Institute for Life Science, Asan Medical Center, Seoul 05505, Korea; Department of Biomedical Engineering, University of Ulsan College of Medicine, Seoul 05505, Korea; Nano Life Science Institute (WPI-NanoLSI), Kanazawa University, Kanazawa 920-1192, Japan; School of Life Science and Technology, Tokyo Institute of Technology, Yokohama 226-8503, Japan; Department of Cell and Developmental Biology, Feinberg School of Medicine, Northwestern University, Chicago, IL 60611 USA; Nano Life Science Institute (WPI-NanoLSI), Kanazawa University, Kanazawa 920-1192, Japan; Faculty of Frontier Engineering, Institute of Science and Engineering, Kanazawa University, Kanazawa 920-1192, Japan; Centre for Microvascular Research, William Harvey Research Institute, Faculty of Medicine and Dentistry, Queen Mary University of London, London E1 4NS, United Kingdom; Centre for Microvascular Research, William Harvey Research Institute, Faculty of Medicine and Dentistry, Queen Mary University of London, London E1 4NS, United Kingdom; Department of Biochemistry, University of Zurich, Zurich 8057, Switzerland; Department of Cell and Developmental Biology, Feinberg School of Medicine, Northwestern University, Chicago, IL 60611 USA; Nano Life Science Institute (WPI-NanoLSI), Kanazawa University, Kanazawa 920-1192, Japan; Faculty of Frontier Engineering, Institute of Science and Engineering, Kanazawa University, Kanazawa 920-1192, Japan; Cell Biology Center, Institute of Innovative Research, Tokyo Institute of Technology, Yokohama 226-8503, Japan; School of Life Science and Technology, Tokyo Institute of Technology, Yokohama 226-8503, Japan; Cell Biology Center, Institute of Innovative Research, Tokyo Institute of Technology, Yokohama 226-8503, Japan; Nano Life Science Institute (WPI-NanoLSI), Kanazawa University, Kanazawa 920-1192, Japan

**Keywords:** lamin, lamina, nuclear envelope rupture, HGPS, nucleus

## Abstract

The nuclear lamina (NL) lines the nuclear envelope (NE) to maintain nuclear structure in metazoan cells. The major NL components, the nuclear lamins contribute to the protection against NE rupture induced by mechanical stress. Lamin A (LA) and a short form of the splicing variant lamin C (LC) are diffused from the nucleoplasm to sites of NE rupture in immortalized mouse embryonic fibroblasts (MEFs). LA localization to the rupture sites is significantly slow and weak compared with LC, but the underlying mechanism remains unknown. In this study, wild-type (WT), Hutchinson–Gilford Progeria syndrome (HGPS) knock-in MEFs expressing progerin (PG, an LA mutant lacking the second proteolytic cleavage site), and LA/C-knockout MEFs transiently and heterogeneously expressing LA/C WTs and mutants fused to mEmerald are examined before and after NE rupture induced by single-cell compression and laser microirradiation. The farnesylation at the CaaX motif of unprocessed LA and the inhibition of the second proteolytic cleavage decrease the nucleoplasmic pool and slow the localization to the rupture sites in a long-time window (60–70 min) after the induction of NE rupture. Our data could explain the defective repair of NE rupture in HGPS through the farnesylation at the CaaX motif of unprocessed progerin. In addition, unique segments in LA-specific tail region cooperate with each other to inhibit the rapid accumulation within a short-time window (3 min) that is also observed with LC.

**Significance Statement**

In mammalian cells, the nuclear envelope (NE) surrounds the nucleus to protect the genetic material from being damaged by mechanical force. The nuclear lamina (NL) lines the NE, and the structural component of the NL, lamin A (LA) is slowly localized to sites of NE rupture compared with lamin C (LC). This study reveals that the CaaX motif, the second proteolytic cleavage site, and LA-specific segments in the C-terminal tail region of LA are critical for localizing to the rupture sites.

## Introduction

In eukaryotic cell nuclei, the nuclear envelope (NE) spatially separates the nuclear genome from the cytoplasm. The barrier function of the NE is due to two phospholipid bilayers of the inner nuclear membrane (INM) and outer nuclear membrane (ONM). The INM is in contact with chromatin at the nuclear side via INM proteins (1), whereas the ONM is connected with the cytoskeletal system through the linker of nucleoskeleton and cytoskeleton (LINC) complex (2), and is continuous with membranous organelles including the endoplasmic reticulum (ER) (3). The nuclear lamina (NL) is closely apposed to the INM and associates with heterochromatin to support nuclear structure. Nuclear pore complexes (NPCs) fill holes formed by the INM-ONM fusion for nucleo-cytoplasmic transport and interact with euchromatin (4).

The NL structure consists of the nuclear lamins and associated proteins (5). The lamins are type V intermediate filament proteins, which are subdivided into A-type lamins (lamins A [LA] and C [LC]) and B-type lamins (lamins B1 [LB1] and B2 [LB2]) (6, 7). LA and LC are derived from the single gene *LMNA* by alternative splicing (8), whereas LB1 and LB2 are encoded by separate genes *LMNB1* and *LMNB2*, respectively (9–12). Lamin molecules assemble into a filament with a diameter of ∼3.5 nm, and these filaments appear to nonrandomly lay on top of each other to form meshworks of the NL with a thickness of ∼13.5 nm in immortalized mouse embryonic fibroblasts (MEFs) (13, 14). LA/C and LB1 filaments associate with the outer ring structure of the NPC and are involved in regulating the distribution of the meshworks and NPCs (13–16).

The CaaX motif at the C terminus of unprocessed LA, LB1, and LB2 (pre-LA, pre-LB1, and pre-LB2, respectively) is post-translationally processed in steps, including the attachment of farnesyl group to the cysteine residue of the CaaX motif by a farnesyltransferase (17, 18), proteolytic cleavage of three residues (−aaX) by aaX endopeptidases (19), and carboxyl methylation of the terminal carboxylic acid group (−COOH) of the C-terminal cysteine residue by a carboxyl methyltransferase (20). The maturation of LB1 and LB2 is completed by these steps. In case of the farnesylated and carboxymethylated pre-LA, an additional 15 C-terminal residues including the farnesylated and carboxymethylated cysteine are cleaved off by zinc metallopeptidase STE24 (Zmpste24/FACE1) to produce the mature form (mature-LA) (21, 22).

**Fig. 1.**
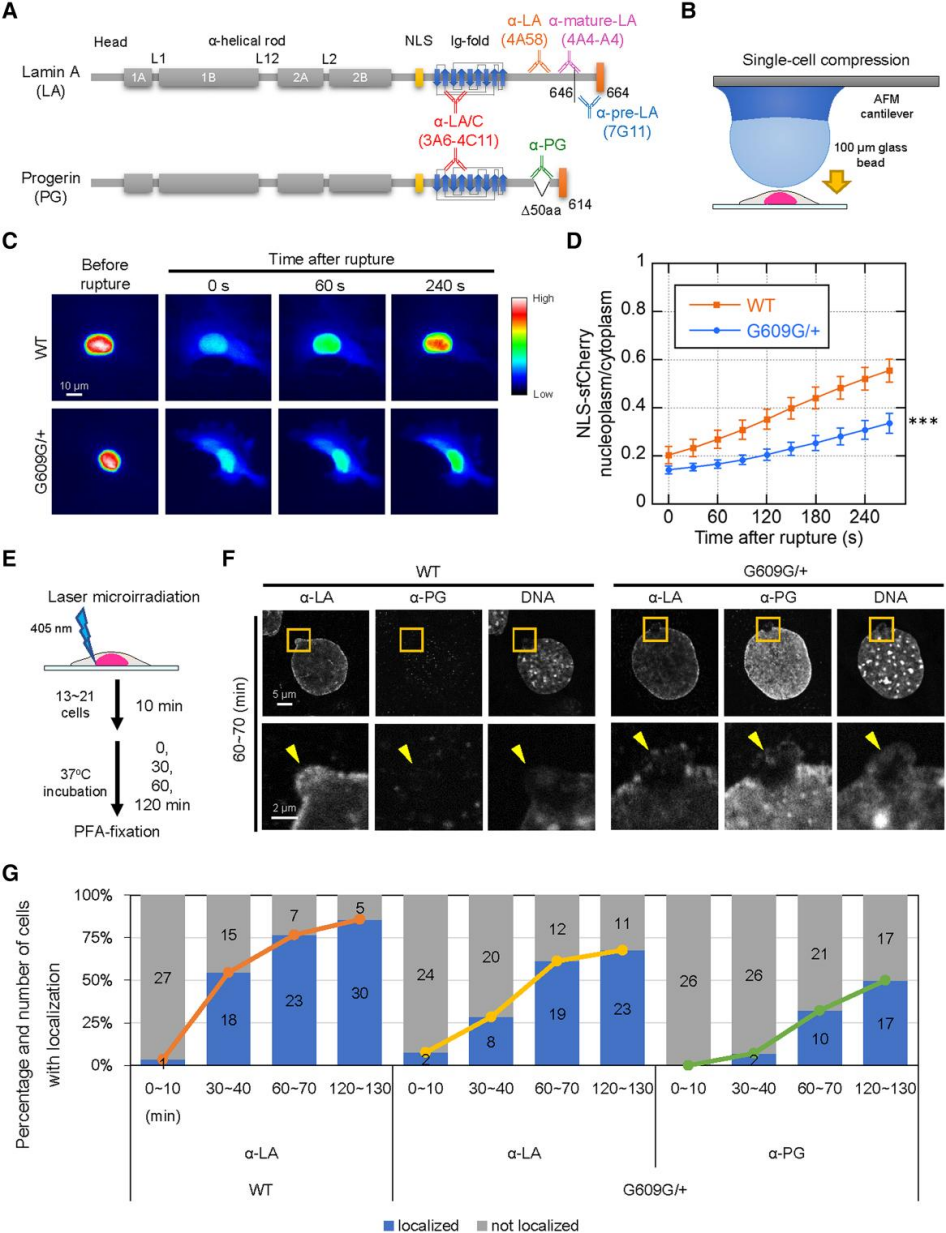
The localization kinetics of LA and PG at the rupture sites in WT and G609G/+ nuclei. A) Protein architecture of LA and PG. The coiled-coil central rod domain (gray), the NLS (yellow), the β-strand comprising the Ig-fold domain (blue), and the CaaX motif (orange) are shown in the diagram. Anti-LA (4A58) and anti-PG do not cross-react with each other. Anti-pre-LA (7G11) recognizes the last amino acids that is proteolytically cleaved off and does not react with PG. Anti-mature-LA (4A4-A4) recognizes mature-LA but not pre-LA. Anti-LA/C (3A6-4C11) recognizes all A-type lamin isoforms. B) NE rupture was induced by single-cell compression with a 100-μm bead attached on a cantilever, as shown in the diagram. C and D) Time-lapse images of NLS-sfCherry expressed in WT and G609G/+ cells were acquired with 30-s intervals for 5 min after the induction of NE rupture by single-cell compression. C) The fluorescence intensities in the cells are shown with rainbow color. Bar: 10 μm. D) The nuclear-to-cytoplasmic intensity (N/C) ratios of NLS-sfCherry were measured, normalized to the initial intensities, and plotted to monitor the nuclear reentry in the cells (means ± SEM; *n* = 20 cells from two independent experiments; ***, *P* < 0.001 from WT by a mixed-effects model). E) NE rupture was induced by 405-nm laser microirradiation with a 2-μm diameter spot at the NE of WT and G609G/+ nuclei. F and G) 13 to 21 of nuclei were laser-microirradiated within 10 min and incubated for 0, 30, 60, and 120 min, followed by fixation with 4% PFA/0.1% Triton-X 100. The fixed cells were immunostained with a combination of the anti-LA and anti-PG, and the DNA was stained with Hoechst 33342. F) Representative confocal images of nuclei in the cells fixed within 60–70 min after the induction of NE rupture by laser microirradiation. Magnified views of the areas indicated with orange boxes are shown (bottom). The rupture sites are indicated with yellow arrowheads (bottom). Bars: 5 μm (top) and 2μm (bottom). G) Percentiles of the cells with (blue) and without (gray) the localization of LA and PG to the rupture sites. The numbers of analyzed cells from two independent experiments are indicated in the bar charts. The line graphs show the kinetics of the percentage of the cells with localization of LA and PG to the rupture sites.

The NE is locally ruptured during physiological and pathological circumstances. Immediately after NE rupture, NE components are recruited to the rupture sites with the endosomal sorting complex required for transport-III (ESCRT-III) complex and barrier-to-autointegration factor (BAF) (23–25). Nuclear DNA adjacent to the rupture sites is recognized by the DNA sensor cyclic GMP-AMP synthase (cGAS) and its downstream signaling effector stimulator of interferon genes (STING) (23–25). The frequencies of spontaneous and forced NE rupture are significantly increased by the depletion of lamins (26–29). Numerous mutations that have been found throughout the *LMNA* gene cause a spectrum of human genetic disorders, collectively called laminopathies (30). Laminopathies associated with dilated cardiomyopathy (DCM), muscular dystrophy (MD), familial partial lipodystrophy (FPLD), and Hutchinson–Gilford progeria syndrome (HGPS) are frequently accompanied by spontaneous NE rupture (31–33). In HGPS, the heterozygous transversion of a nucleotide substitution at position 1824 C > T (G608G) leads to abnormal splicing of the last 150 nucleotides of exon 11, resulting in the production of unprocessed HGPS mutant, progerin (pre-PG) which lacks 50 amino acids (residues 607–656) from the C terminus of pre-LA (Fig. 1A). Because these residues contain the second proteolytic cleavage site involved in pre-LA processing, the mature form (mature-PG) remains farnesylated and carboxymethylated, leading to its permanent association with the INM (34, 35).

Previous studies demonstrate that LA/C is recruited to the rupture sites with BAF (36–38). LA is significantly slow and weak in localizing to the rupture sites compared with LC (38–40). However, the underlying mechanism for slowing its localization to the rupture sites remains to be elucidated. Here, we investigate the role of the C-terminal tail region specific for LA in this mechanism. We previously showed that mEmerald-fused LA accumulated at the rupture sites only when it was overexpressed at a high level (38), and the accumulation kinetics appeared to be artifactual because it was not confirmed with endogenous LA detected by immunofluorescence (38). Therefore, we establish the ectopic expression system for live-cell imaging and fluorescence correlation spectroscopy (FCS) to express mEmerald-fused LA, alternate forms of LA, and LC at a low level with the subcloned endogenous promotor in wild-type (WT), HGPS, and LA/C-knockout (KO) MEFs. Our data reveal the significance of the LA-specific tail region for retaining its small pool in the nucleoplasm and slowing its localization to the rupture sites.

## Results

### PG is not only slow in localizing to the rupture sites but also slows LA localization

To investigate the involvement of the pre-LA processing in retaining small pool of LA in the nucleoplasm and slowing its localization to the rupture sites, we used immortalized WT MEFs and heterozygous knock-in MEFs with PG expression from a mutant allele of the *Lmna* gene carrying the c.1827C > T; p.Gly609Gly mutation (*Lmna^G609G/+^*, G609G/+). This mutation is equivalent to the HGPS mutation in the human *LMNA* gene (41, 42). These cells stably expressed super-folder Cherry harboring two NLSs derived from SV40 large T antigen and c-Myc (NLS-sfCherry) as an NE rupture marker (38). Because spontaneous NE rupture is frequently observed in skin fibroblasts derived from a HGPS patient and mouse smooth muscle cells (SMCs) expressing PG (31, 33), we first examined whether G609G/+ MEFs also exhibit deformed nuclei, the leakage of NLS-sfCherry to the cytoplasm, the mis-localization, or decreased expression of LB1, which are indicative of spontaneous NE rupture and nuclear blebbing (15, 43). WT and G609G/+ cells were stained with Hoechst 33342 for DNA and specific antibodies directed against LB1 (Fig. S1A). Unlike the HGPS patient cells and PG-expressing mouse SMCs, G609G/+ cells did not show these phenotypes compared with WT cells (Table S1 and Fig. S1B), indicating that the HGPS mutation is not accompanied by spontaneous NE rupture in these cells. Next, to examine the effect of this mutation on recovery from forced NE rupture, single cells were compressed with a 100-μm glass bead attached on a cantilever, and then, the nuclear reentry of NLS-sfCherry was observed by time-lapse imaging before and after the induction of the rupture (Fig. 1B) (44). This method was confirmed by the expression of super-folder GFP (sfGFP)-fused cGAS in these cells as the leakage of NLS-sfCherry to the cytoplasm was accompanied by the accumulation of cGAS-sfGFP at the rupture sites (Fig. S1C and Movies S1 and S2). Interestingly, G609G/+ cells showed a significantly slow reentry compared with the WT cells which had a ∼50% recovery from the original fluorescence intensity ∼4 min after the induction of NE rupture (Figs. 1C, D and S1C and Movies S1 and S2). These results indicate that PG expression slows the recovery from forced NE rupture.

To determine the localization kinetics of LA and PG at the rupture sites within a relatively long-time window, WT and G609G/+ cells were fixed at 0–10, 30–40, 60–70, and 120–130 min after the induction of NE rupture by 405-nm laser microirradiation (Fig. 1E) (38) and then stained with Hoechst 33342 for DNA and the specific antibodies directed against LA and PG which do not cross-react to each other (Fig. 1A). At 0–10, 30–40, 60–70, and 120–130 min after the rupture, LA was localized to the rupture sites in ∼4, ∼55, ∼77, and ∼86% of WT nuclei and ∼8, ∼29, ∼61, and ∼68% of G609G/+ nuclei, respectively (Figs. 1F, G and S1D), whereas PG was slower in localizing to the rupture sites of G609G/+ nuclei than LA, showing ∼0, ∼7, ∼32, and ∼50%, respectively (Figs. 1F, G and S1D). These results suggest that PG expression decelerates the localization kinetics of LA at the later time points (60 and 120 min). To exclude the possibility that the immortalization process may affect the localization of LA and PG to the rupture sites, primary WT and G609G/+ MEFs were examined 60–70 min after the induction of NE rupture by laser microirradiation. LA was localized to the rupture sites in ∼55 and ∼45% of these WT and G609G/+ nuclei, respectively, and PG was in 29% of G609G/+ nuclei (Fig. S1E and F). These values were relatively small compared with immortalized WT and G609G/+ cells, respectively (Fig. 1G and S1F). However, PG is still slower in localizing to the rupture sites than LA regardless of the immortalization process. In addition, both LA and PG are likely to be recruited to the rupture sites with BAF within a relatively long-time window according to our results from BAF knockdown (KD) experiments with short hairpin RNAs (shRNAs) (Fig. S1G–I). WT cells expressing a shRNA for the control (Scramble) or two shRNAs (shBAF #1 and #2) were fixed at 60–70 min after the induction of NE rupture by laser microirradiation, and then, LA localization was determined by immunofluorescence. As expected, LA was localized to the rupture sites in ∼61, 10, and ∼16% of the control and BAF-KD#1 and #2 nuclei, respectively (Fig. S1H and I), indicating that BAF is required for LA localization to the rupture sites. These results are similar to a previous finding to indicate that BAF rapidly recruits overexpressed GFP-LA to the rupture sites (36).

### PG slows LC accumulation to the rupture sites

Because the accumulation of LC to the rupture sites was much earlier (within 3 min) than the localization of LA or PG (30–120 min) (38–40) (Fig. 1F and G) and LC depletion accelerates the cytoplasmic leakage of NLS-sfCherry at the early time points (within 10 min) (38), LC might be directly linked to the delay in retrieving NLS-sfCherry from the cytoplasm of G609G/+ cells within 10 min after the induction of forced NE rupture (Fig. 1C and D). Therefore, PG expression could slow LC accumulation to the rupture sites. To test this idea, endogenous LA, LC, and PG were directly labeled with a LA/C-specific genetically encoded probe, designed ankyrin repeat protein (DARPin)-LaA_6 (45), fused with sfGFP, sfGFP-DARPin-LA6 in WT and G609G/+ cells expressing NLS-sfCherry (38) (Fig. S2A). Then, live-cell imaging was carried out before and after the induction of NE rupture by laser microirradiation. Because endogenous LA and PG did not localize to the rupture sites within 3 min after the induction of NE rupture (38) (Figs. 1G and S1D), sfGFP-DARPin-LA6 accumulating at the rupture sites only represented endogenous LC within such a short-time window. Immediately after the induction of NE rupture, the accumulation of sfGFP-DARPin-LA6 was significantly weaker in G609G/+ nuclei compared with WT nuclei (Fig. S2B and C), indicating that PG expression attenuates LC accumulation.

### PG slows the diffusion of LC in the nucleoplasm

Because of the slow recruitment of LC from the nucleoplasm to the rupture sites in G609G/+ nuclei (Fig. S2B and C), the diffusion fractions and coefficients in the nucleoplasm could be decreased in G609G/+ nuclei compared with WT nuclei. To determine them, mEmerald-LC was stably expressed at a low level from the *Rosa26* locus under the endogenous *Lmna* promotor in WT and G609G/+ cells, and FCS was performed using these cells (Fig. S3A–C). The auto-correlation curve obtained by the signal detection was fitted with fast and slow components (15, 46, 47). Interestingly, the diffusion coefficients of fast components were significantly high in WT nuclei compared with G609G/+ nuclei (Fig. S3D). This result indicates that PG expression significantly decreases the diffusion coefficient of fast component despite the increased fraction, but this degree of difference is insufficient for explaining the weak accumulation of sfGFP-DARPin-LA6 at the rupture sites in G609G/+ nuclei compared with WT nuclei.

### The nucleoplasmic pools of LA and PG are increased by the inhibition of farnesylation

**Fig. 2.**
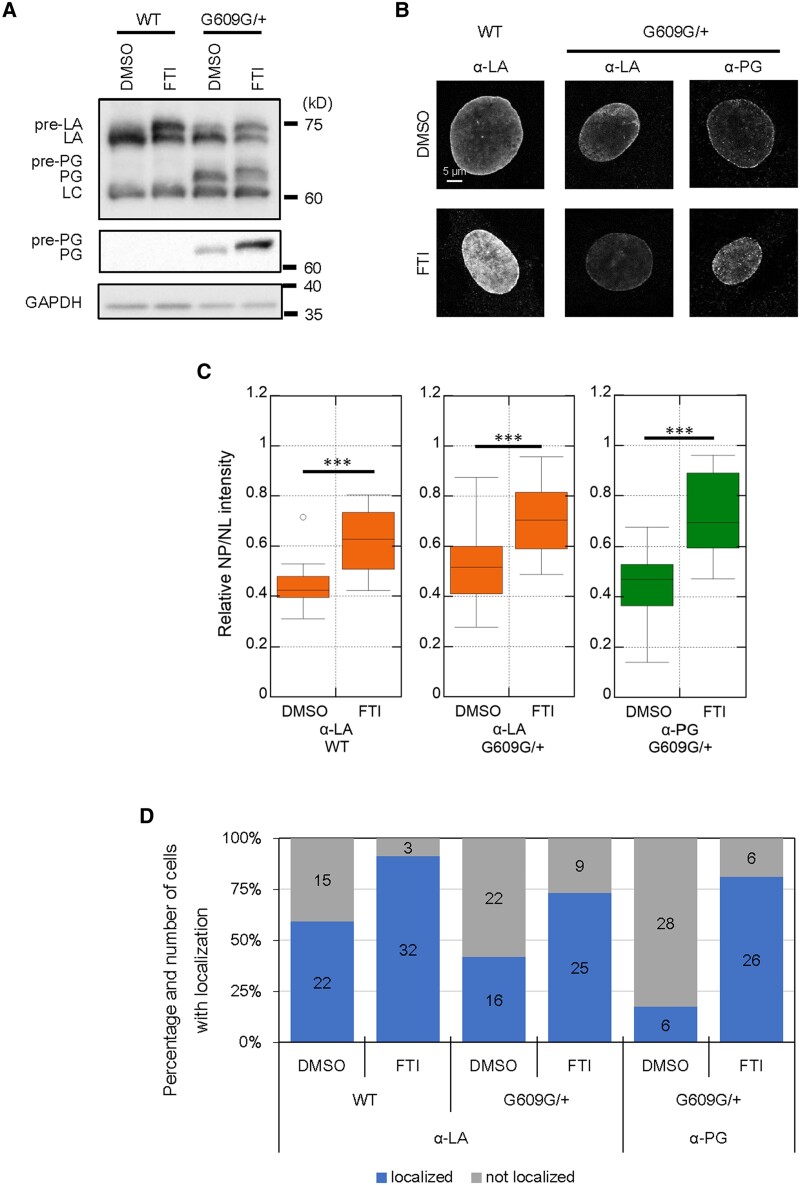
Localization of LA and PG to the rupture sites in DMSO or FTI-treated WT and G609G/+ nuclei. A) Whole-cell lysates from WT and G609G/+ cells treated with 0.1% DMSO or 3.2 μM lonafarnib (FTI) were probed with anti-LA/C (3A6-4C11), anti-PG, and anti-GAPDH (as loading control) for immunoblotting. Positions of the size standards are shown on the right. B and C) The indicated cells were immunostained with the anti-LA (4A58) and anti-PG. B) Representative microscopic images of single confocal sections. Bar: 5 μm. C) Ratios of the average fluorescence intensity in the nucleoplasmic pool relative to that of the NL (NP/NL ratios) of LA and PG were measured based on immunofluorescence (*n* = 20 cells from two independent experiments; ***, *P* < 0.001 by Welch's t tests). D) 15 to 19 of nuclei were laser-microirradiated and incubated for 60 min, followed by fixation with 4% PFA/0.1% Triton-X 100. The fixed cells were immunostained with the anti-LA and anti-PG. Percentiles of the cells with (blue) and without (gray) the localization of LA and PG at the rupture sites. The numbers of analyzed cells from two independent experiments are indicated in the bar charts.

When the CaaX motifs of pre-LA and pre-PG are farnesylated (18, 35, 48), they are retained at the NE through the hydrophobic interaction with the lipid bilayer of the INM (48, 49). Therefore, the inhibition of farnesylation with FTI could increase their nucleoplasmic pools, leading to their fast localization to the rupture sites. To test this possibility, WT and G609G/+ cells were treated with DMSO for the control or farnesyltransferase inhibitor (FTI), 3.2 µM lonafarnib for 48 h. The expression levels and localization of LA and PG were determined by immunoblotting using the specific antibodies directed against LA/C and by immunofluorescence using the LA and PG antibodies, respectively (Fig. 2A and B). For immunoblotting, pre-LA, mature-LA, pre-PG, mature-PG, and LC were detected with the antibodies directed against LA/C (for all) and PG (for pre- and mature-PG) (Fig. 2A). For immunofluorescence, the LA antibody (for pre- and mature-LA) and the PG antibody (for pre- and mature-PG) were used (Fig. 2B). As expected, the levels of pre-LA and pre-PG were increased in FTI-treated cells compared with the control cells as shown in the upper and lower bands of LA and PG (Fig. 2A). Based on ratios of the average fluorescence intensity in the nucleoplasmic pool relative to that of the NL (NP/NL ratios), LA and PG were significantly abundant in the nucleoplasm of FTI-treated nuclei compared with the control nuclei (Fig. 2B and C), indicating that LA and PG are increased in the nucleoplasmic pool by FTI treatment. Next, these cells were fixed within 10 min and at 60–70 min after the induction of NE rupture by laser microirradiation, and the localization of LA and PG to the rupture sites was detected by immunofluorescence. LA was localized to the rupture sites within 10 min in ∼7 and ∼7% of WT and G609G/+ nuclei for the control and ∼18 and ∼17% of WT and G609G/+ nuclei treated with FTI, respectively, and PG in 0 and ∼37% of the control and FTI-treated G609G/+ nuclei, respectively (Fig. S4A and B). At 60–70 min, LA localization to the rupture sites was observed in ∼59 and ∼42% of the control nuclei and ∼91 and ∼74% of FTI-treated WT and G609G/+ nuclei, respectively, and PG localization in ∼18 and ∼81% of the control and FTI-treated G609G/+ nuclei, respectively (Figs. 2D and S4C). Because FTI treatment is also known to inhibit the farnesylation of B-type lamins (50), the effects on the localization of LA and PG to the rupture sites could also be confounded by altered interactions between A- and B-type lamins. To check the possibility, the localization of LB1 was determined in these cells by immunofluorescence. The NP/NL ratio of LB1 was significantly increased in the nucleoplasm of FTI-treated nuclei compared with the control nuclei (Fig. S4D and E). ∼56 and ∼65% of WT and G609G/+ nuclei treated with FTI showed LB1 localization to the rupture sites at 60–70 min, but ∼3 and ∼3% in the control nuclei, respectively (Fig. S4C and F). These results are similar to those which were observed using GFP-LB1 expressing cells within the short-time window (37). These results indicate that the inhibition of farnesylation promotes the localization of LA, PG, and LB1 to the rupture sites.

### PG becomes diffusible in the nucleoplasm by FTI treatment

**Fig. 3.**
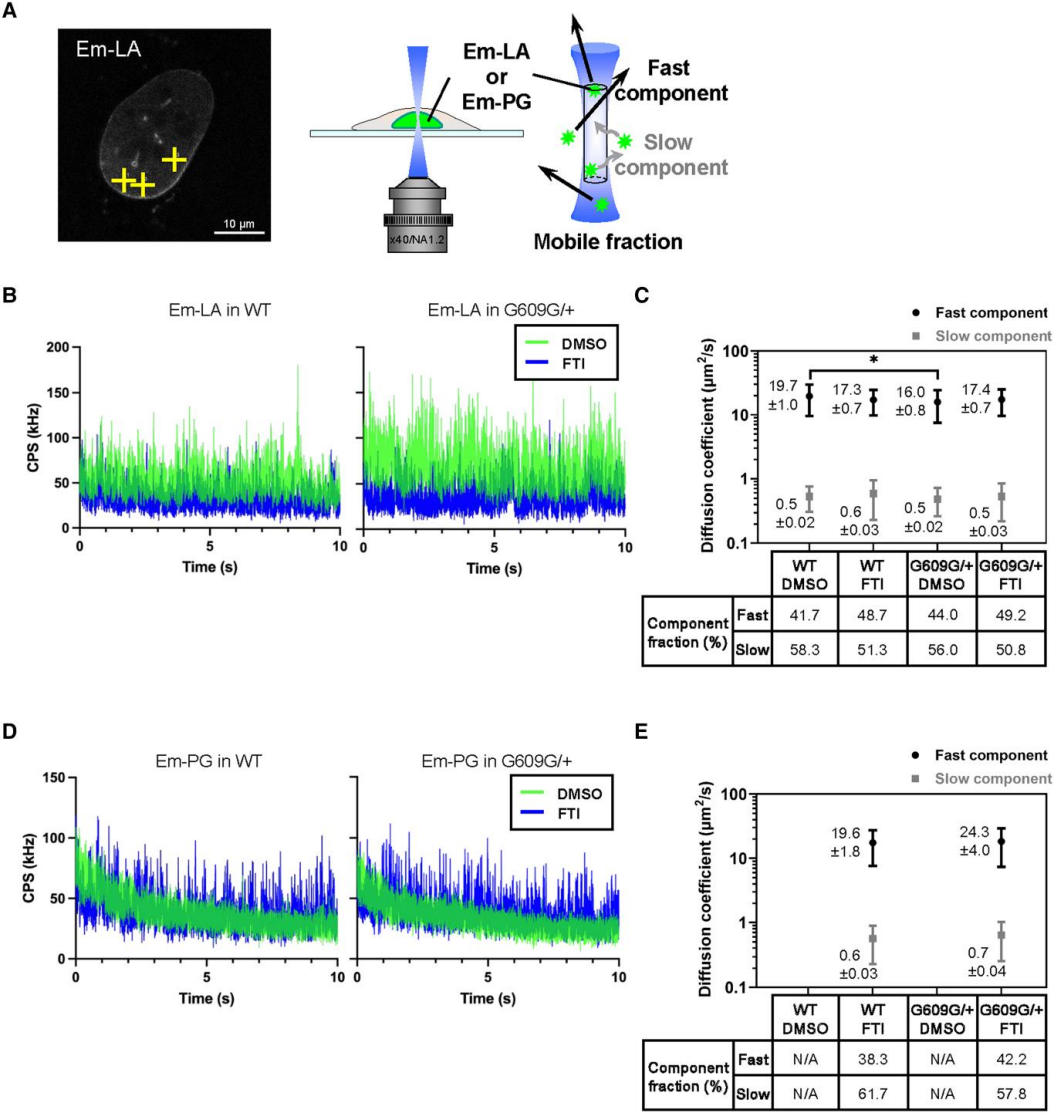
The diffusion kinetics of LA and PG in the nucleoplasm of DMSO- or FTI-treated WT and G609G/+ nuclei. The diffusion fractions of mEmerald-LA (Em-LA) and mEmerald-PG (Em-PG) in DMSO- or FTI-treated WT and G609G/+ nuclei were measured by FCS. A) A representative confocal image of a WT nucleus with Em-LA before FCS measurements. The yellow crosses indicate the points measured by FCS (left). Bar: 10 µm. Em-LA or Em-PG molecules move in or out of the confocal volume (white-out cylinder region in blue) at different speeds, as shown in the diagram (right). B) Representative fluorescence fluctuations of Em-LA in DMSO-treated (green line) and FTI-treated (blue line) of WT and G609G/+ nuclei are plotted by count per second (CPS). C) Diffusion coefficients of fast component (plotted in black) and slow component (plotted in gray) for Em-LA in the cells. D) Representative fluorescence fluctuations of Em-PG in DMSO- (green line) and FTI-treated (blue line) of WT and G609G/+ nuclei are plotted by CPS. E) Diffusion coefficients of fast component (plotted in black) and slow component (plotted in gray) for Em-PG in the cells. C and E) Mean ± SEM are indicated on the left to plots; *n* = 10 cells from two independent experiments; *, *P* < 0.05 by a Games–Howell test. Percentiles of these component fractions are indicated at the bottom of the graph. N/A, not applicable because of photobleaching.

Because the nucleoplasmic pools of LA and PG were increased by FTI treatment (Fig. 2B and C), the nonfarnesylated forms could be highly mobile in the nucleoplasm of FTI-treated cells. To test this idea, the diffusion coefficients of mEmerald-LA and PG were measured by FCS (Fig. 3A). For the FCS measurements, mEmerald-LA and PG were stably expressed at a low level by the same system as described above with mEmerald-LC (Fig. S3A and B), followed by the treatment with DMSO for the control or FTI (Fig. S5). The diffusion fractions of mEmerald-LA in the control and FTI-treated WT and G609G/+ nuclei were all similar to each other (Fig. 3B and C), but the diffusion coefficient of fast component was slightly but significantly small in G609G/+ nuclei compared with WT nuclei, whereas that of slow component remained unchanged (Fig. 3C). These results show that PG expression slows the diffusion speed of mEmerald-LA. On the other hand, the diffusion signals of mEmerald-PG were barely detectable in the nucleoplasm of WT and G609G/+ nuclei for the control. Instead, the signal decays caused by photobleaching were observed during the FCS measurements (Fig. 3D), strongly suggesting that these signals originate from the NL where mEmerald-PG is immobile (15, 51). When WT and G609G/+ cells were treated with FTI, the diffusion signals were detected by FCS as expected (Fig. 3D and E), indicating that PG becomes diffusible in the nucleoplasm by FTI treatment. Therefore, the nucleoplasmic pool is the likely source of PG localized to the rupture sites (Fig. 2C and D).

### The second proteolytic cleavage is required for the localization of LA to the rupture sites

The localization of PG to the rupture sites was significantly slow compared with LA (Fig. 1G), and the permanent association of PG with the INM due to the absence of the second proteolytic cleavage site could decelerate the localization kinetics (34, 35). To check this possibility, Zmpste24, which endoproteolytically processes both the CaaX motif and the second cleavage site (52), was knocked down by shRNAs in WT MEFs, and then, the expression levels and localization of pre- and mature-LA were determined in cells expressing shRNAs for the control or Zmpste24 KD by immunoblotting and immunofluorescence, respectively (Fig. S6A). A specific antibody directed against pre-LA used for immunoblotting and immunofluorescence reacts with pre-LA but not mature-LA (Fig. 1A). The C-terminal residues of pre-LA including the CaaX motif remained partially uncleaved by Zmpste24 KD, resulting in the significant increase and decrease in expression level of pre- and mature-LA, as shown in the upper and lower bands, respectively (Fig. S6A). These results are consistent with the previous findings to indicate that Zmpste24 KD inhibits the second cleavage (53). LA and pre-LA were predominantly localized to the NL, and the NP/NL ratios remained unchanged in Zmpste24-KD cells compared with the control cells (Fig. S6B and C). These cells were fixed at 60–70 min after the induction of NE rupture by laser microirradiation, and then, the localization of LA and pre-LA to the rupture sites was determined by immunofluorescence. LA was localized at the rupture sites in ∼75% of the control cells, whereas LA and pre-LA were completely absent from the rupture sites in Zmpste24-KD cells (Fig. S6D and E), indicating that the second cleavage is required for their localization to the rupture sites.

### Inhibition of farnesylation at the CaaX motif is sufficient for the localization of LA to the rupture sites without the second proteolytic cleavage

**Fig. 4.**
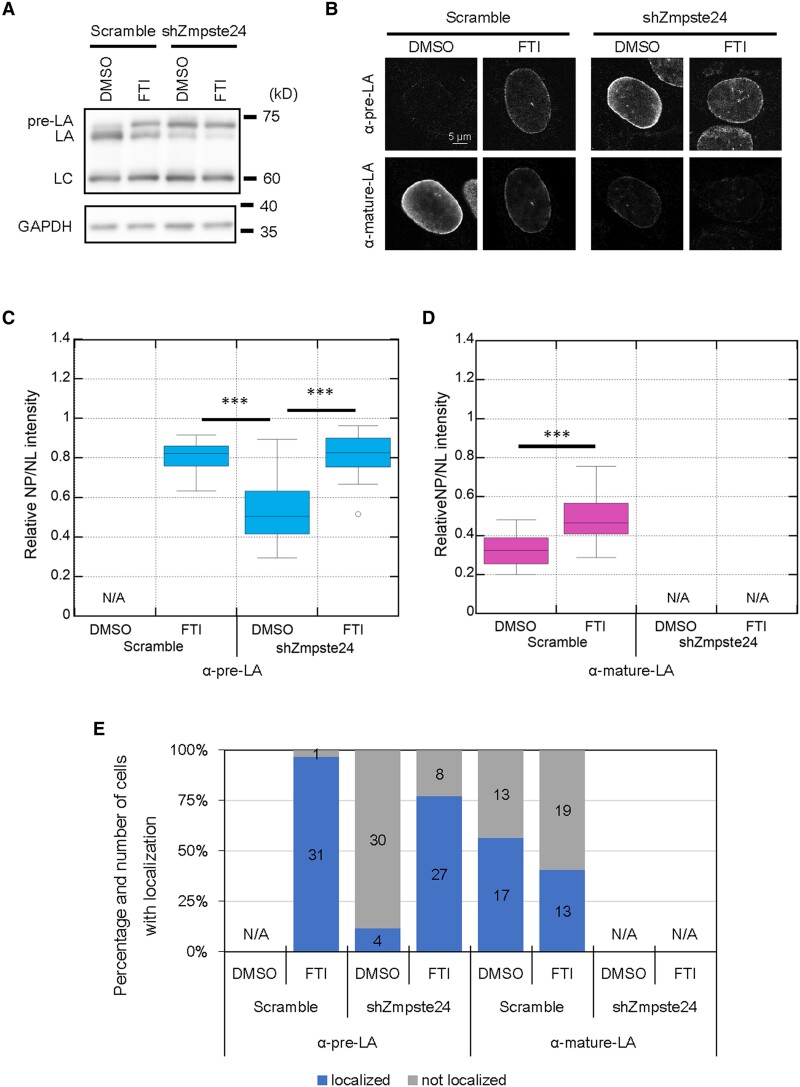
The localization of pre- and mature-LA in the control and Zmpste24-KD WT MEFs treated with DMSO or FTI. A) Whole-cell lysates from the control and Zmpste24-KD cells treated with DMSO or FTI were probed with anti-LA/C (3A6-4C11) and anti-GAPDH (as loading control). Positions of the size standards are shown on the right. B) The control and Zmpste24-KD cells treated with DMSO or FTI were immunostained with a combination of anti-pre-LA (7G11) and anti-mature-LA (4A4-A4). Bar: 5 μm. NP/NL ratios of pre-LA (C) and mature-LA (D) in the cells were measured based on immunofluorescence (*n* = 20 cells from two independent experiments; ***, *P* < 0.001 by Games–Howell tests for pre-LA and by Welch's t test for mature-LA). E) 15 to 18 of nuclei were laser-microirradiated within 10 min and incubated for 60 min, followed by fixation with 4% PFA/0.1% Triton-X 100. The fixed cells were immunostained with a combination of the anti-pre-LA and anti-mature-LA. Percentiles of the indicated cells with (blue) and without (gray) the localization of LA and pre-LA to NE rupture sites. The numbers of analyzed cells from two independent experiments are indicated in the bar charts. C–D) N/A, not applicable due to below the detection limit.

Zmpste24 depletion significantly increased the pre-LA expression level (54) (Fig. S6A and B), which is accompanied by the farnesylation at the CaaX motif (55). Therefore, the inhibition of the farnesylation with FTI could release the pre-LA from the NE, resulting in the fast localization of pre-LA to the rupture sites. To test this idea, the control (Scramble) and Zmpste24-KD cells were treated with DMSO or FTI as before, and the expression levels and localization of pre- and mature-LA were determined by immunoblotting and immunofluorescence, respectively (Fig. 4A and B). As expected, Zmpste24 KD increased and decreased the pre- and mature-LA levels, respectively, in DMSO- and FTI-treated cells (Fig. 4A). Zmpste24 KD and FTI treatment were similar in action to the control cells, but the synergetic effect was very subtle (Fig. 4A). To analyze the localization of pre- and mature-LA in those cells by immunofluorescence, specific antibodies directed against them that do not cross-react with each other were used. In the control cells treated with DMSO, pre-LA was under the detection limit, whereas mature-LA was predominantly localized to the NL (Fig. 4B–D). When the control cells were treated with FTI, pre-LA became clearly visible in the NL and mature-LA was less prominent there instead (Fig. 4B–D). In Zmpste24-KD cells treated with DMSO, pre-LA was localized to the NL and mature-LA was undetectable (Fig. 4B–D). FTI treatment decreased pre-LA localization to the NL up to the level of the control cells treated with FTI and mature-LA was undetectable (Fig. 4B–D). These results were consistent with the known effect of FTI treatment, which inhibit the farnesylation at the CaaX motif and the following processes including the second cleavage, leading to release the pre-LA from the NE (56). As FTI treatment increases nucleoplasmic LA levels regardless of the second cleavage, we hypothesize that LA localizes to the rupture site more efficiently in FTI. The localization of pre- and mature-LA to the rupture sites was determined in these cells by immunofluorescence at 60–70 min after the induction of NE rupture by laser microirradiation. Pre-LA was localized to the rupture sites in ∼97, ∼12, and ∼77% of FTI-treated control, DMSO- and FTI-treated Zmpste24-KD nuclei, respectively (Figs. 4E and S7). This indicates that pre-LA without farnesylation present in the nucleoplasm effectively localizes to the rupture sites, supporting the hypothesis. Despite the increased localization of pre- and mature-LA to the rupture sites by FTI treatment (Fig. 2D), the localization of mature-LA was slightly decreased in the control cells (Fig. 4E), probably because its nucleoplasmic pool was not as abundant as pre-LA (Figs. 2C and 4C and D).

### LA differs from LC in localization to the rupture sites through two specific sequences in the tail region

**Fig. 5.**
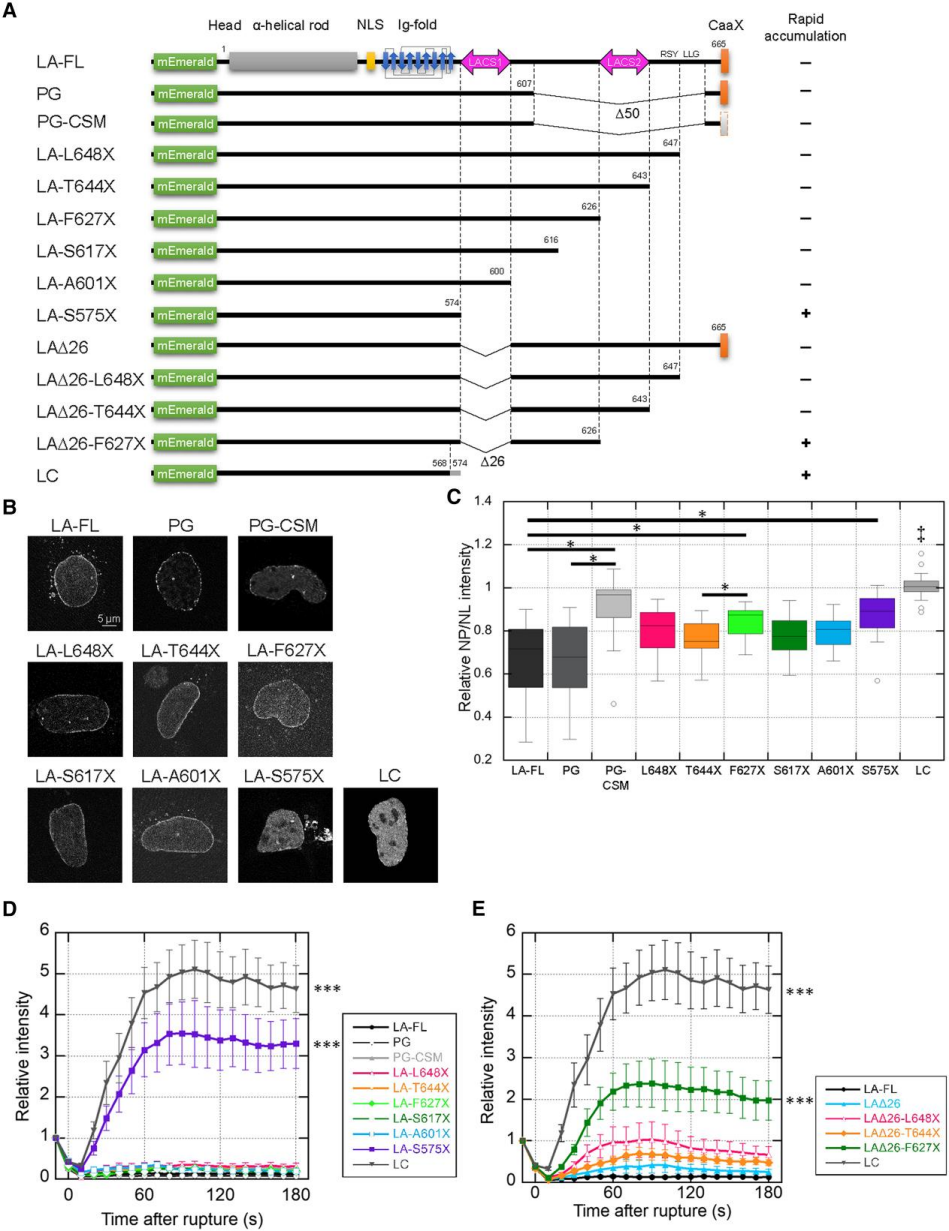
Rapid accumulation of LA truncation and PG deletion mutants at the rupture sites in LA/C-KO nuclei. A) Architecture of mEmerald-fused LA-full-length (FL), PG, PG-CSM, L648X, T644X, F627X, S617X, A601X, S575X, LAΔ26, LAΔ26-L648X, LAΔ26-T644X, LAΔ26-F627X, and LC. Terminal amino acids are numbered, and their accumulation dynamics are summarized on the right (+, accumulated at the rupture site; −, not accumulated). B and C) The mEmerald-fused LA-FL, PG, PG-CSM, L648X, T644X, F627X, S617X, A601X, S575X, and LC were ectopically expressed under the *Lmna* promoter in LA/C-KO cells. B) Representative confocal images of the indicated nuclei. Bar: 5 μm. C) The NP/NL ratios were measured based on mEmerald signals (*n* = 15–20 cells from two independent experiments; ‡, *P* < 0.001 compared with others except PG-CSM and *, *P* < 0.05 by a Games–Howell test). D and E) Time-lapse images of mEmerald-fused LA-FL, LA mutants, PG, PG-CSM, and LC were acquired with 10-s intervals for 3 min after the induction of NE rupture by laser microirradiation, and the relative intensities at the rupture sites are plotted in the graphs. The fluorescence intensities at the rupture sites were measured and normalized to the initial intensities (means ± SEM; *n* = 20 cells from two independent experiments; ***, *P* < 0.001 from LA-FL by a mixed-effects model). D) mEmerald-fused LA-FL, PG, PG-CSM, L648X, T644X, F627X, S617X, A601X, S575X, and LC. E) mEmerald-fused LA-FL, LAΔ26, LAΔ26-L648X, LAΔ26-T644X, and LAΔ26-F627X. LA-FL and LC are reproductions of D.

The inhibition of farnesylation at the CaaX motif of PG significantly increased the nucleoplasmic pool and promoted the localization to the rupture sites within 10 min after the induction of NE rupture (Fig. S4A and B). Interestingly, PG was not localized to the rupture sites within the first half of the time window (0–5 min), whereas LC rapidly accumulated (38) (Fig. S2). Therefore, we confirmed the effect of this inhibition on their NP/NL ratios and the accumulation kinetics at the rupture sites within the short-time window (3 min) by live-cell imaging using mEmerald-fused LA-full length (FL), LC, PG, and the nonfarnesylated mutant of PG, PG-CSM that lacks the isoleucine within the CaaX motif (Fig. 5A). We used LA/C-KO cells to express these LA/C WTs and mutants. Because these cells do not express endogenous LA/C, this approach allows us to avoid complications from the interactions of these mutants with endogenous LA/C. In addition, they were transiently and heterogeneously expressed at a low level with the subcloned endogenous promotor in LA/C-KO cells so as not to cause artifacts by the overexpression. As expected, the NP/NL ratio of mEmerald-PG was similar to that of mEmerald-LA-FL, whereas that of mEmerald-PG-CSM was significantly high but not to the extent of mEmerald-LC (Fig. 5B and C). mEmerald-PG and mEmerald-PG-CSM did not accumulate at the rupture sites (Fig. 5D), indicating that the inhibition of the farnesylation is insufficient for exhibiting the rapid accumulation that was similar to that of mEmerald-LC. These results strongly suggest that the tail regions specific for LA and PG are involved in inhibiting the rapid accumulation. In humans and mice, LC harbors identical six amino acids, whereas LA has 98 and 97 amino acids, following the residues 1–566 and 1–568 shared between LA and LC, respectively (Fig. S8A). Because these LC-specific amino acids do not contribute to the rapid accumulation at the rupture sites (38), mEmerald fusion proteins of LA truncation mutants, LA-S575X, LA-A601X, LA-S617X, LA-F627X, LA-T644X, and LA-L648X, were tested for their NP/NL ratios and the accumulation kinetics within 3 min (Fig. 5A). Among these mutants, mEmerald-LA-S575X has the highest NP/NL ratio (Fig. 5B and C), and only this mutant rapidly accumulated within 3 min (Figs. 5D and S8B), indicating that the residues 575–600 are involved in inhibiting the rapid accumulation. Because the deletion mutant mEmerald-LAΔ26 did not accumulate at the rupture sites (Fig. 5E), further truncation was performed at the C-terminal side of mEmerald-LAΔ26 to produce mEmerald-LAΔ26-F627X, mEmerald-LAΔ26-T644X and mEmerald-LAΔ26-L648X (Fig. 5A). The NP/NL ratio remained unchanged by these truncations compared with mEmerald-LAΔ26 (Fig. S8C and D), but only mEmerald-LAΔ26-F627X rapidly accumulated (Figs. 5E and S8E). The deletion of the residues 575–600 from PG might also accelerate the localization kinetics at the rupture sites because it does not have the residues 627–643. However, PGΔ26 did not accumulate at the rupture sites probably due to the farnesylation at the CaaX motif (Fig. S8F). These results show that the residues 627–643 assist LAΔ26 to inhibit the rapid accumulation without decreasing the nucleoplasmic pool. We therefore designated the residues 575–600 as LA-characteristic sequence-1 (LACS1) and the residues 627–643 as LACS2.

## Discussion

**Fig. 6.**
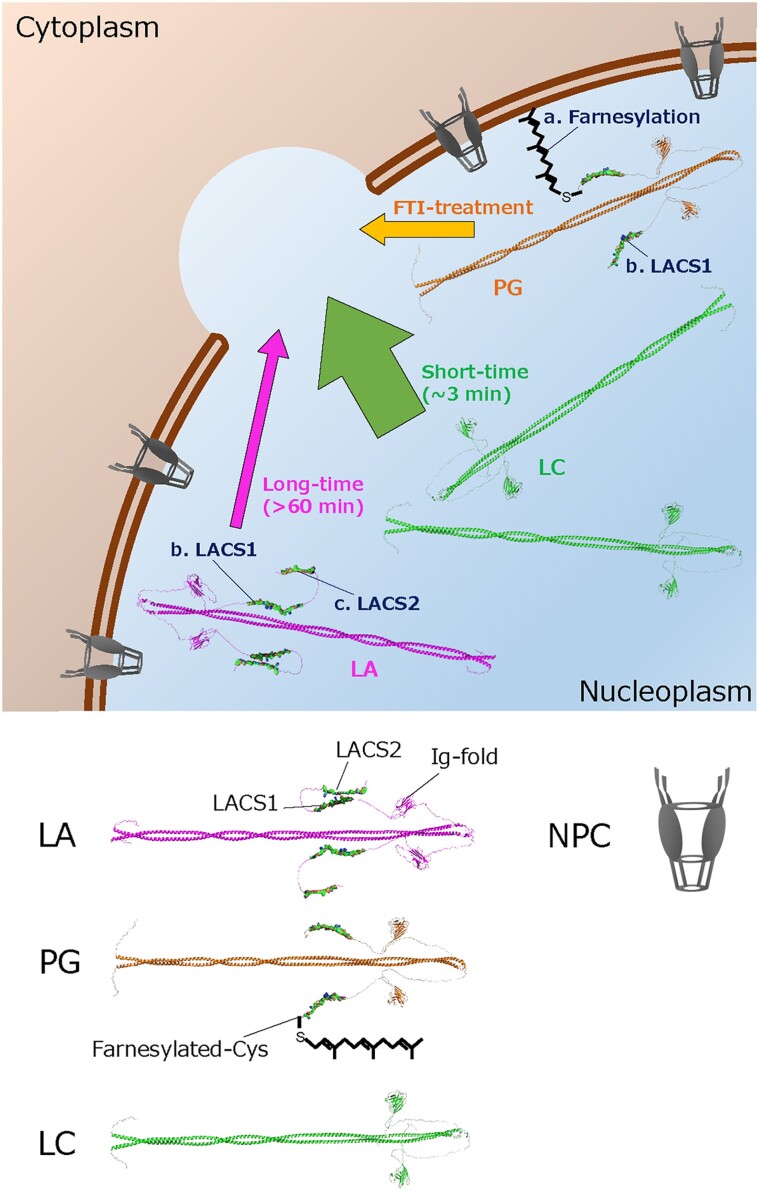
Schematic diagram of the difference in localization to the rupture sites between LA, LC, and PG. LA (magenta) is slowly localized to the rupture sites compared with LC (green) because it contains two LACSs in the specific tail region. PG (orange) is even slower in localizing to the rupture sites than LA because of the permanent association with the INM. PG expression also slows the recruitment of LA and LC to the rupture sites. When the farnesylation of PG is inhibited by FTI treatment, the localization kinetics becomes similar to that of LA. Structural images of lamin homodimers were predicted by AlphaFold2 (61) and edited by the PyMOL (Schrödinger).

Although our results indicate differences in cell culture between LC and LA, the biological significance is less clear because mouse models show that the synthesis of LC is dispensable in laboratory mice (57–59), and conversely mice that produce LC but no LA or pre-LA are entirely healthy (59, 60). Our previous study demonstrates that LC but not LA rapidly accumulates at the rupture sites within the short-time window (3 min) after the induction of NE rupture because LC is more abundant in the nucleoplasm than LA (38–40). On the other hand, LA is slowly and weakly localized to the rupture sites within the long-time window (60–70 min) (38–40). The recruitment of both LA and LC to the rupture sites requires both the immunoglobulin-like fold (Ig-fold) domain that binds to BAF and NLS, and it is not attributable to the LC-specific amino acids (36, 38). In this study, we analyze the LA-specific tail region to determine a molecular mechanism for retaining its small pool in the nucleoplasm and decelerating localization kinetics at the rupture sites, and Fig. 6 illustrates our current findings following our previous study (38). The proteolytic removal of 15 amino acids containing the farnesylated C-terminal end of pre-LA by Zmpste24 increases the nucleoplasmic pool and accelerates the localization kinetics at the rupture sites within the long-time window after the induction of NE rupture (Fig. 6a). In HGPS, pre-PG lacks 50 amino acids containing the second proteolytic cleavage site from the C terminus of pre-LA, leading to the permanent association with the INM, which significantly decreases the nucleoplasmic pool and decelerates the localization kinetics at the rupture sites. In contrast, FTI treatment pronouncedly increases the nucleoplasmic pools of LA and PG and promotes their localization to the rupture sites. Therefore, the processes for increasing their nucleoplasmic pools are likely to be rate-limiting steps for the localization kinetics within this time window. Furthermore, LACS1 cooperates with LACS2 to inhibit the rapid accumulation at the rupture sites within the short-time window (Fig. 6b and c).

We use immortalized WT and G609G/+ MEFs to analyze the NP/NL ratios of LA, pre-LA and PG, and their localization kinetics at the rupture sites. Unlike HGPS patient cells and mouse SMCs expressing PG (31, 33, 43), our G609G/+ cells do not exhibit the formation of deformed nuclei and spontaneous NE rupture, which could be due to the immortalization process with SV40 large T antigen. In oncogene-induced senescence of normal human fibroblasts, DNA protrusion from the nucleus, micronuclei formation, and the leakage of nuclear contents are coupled to the loss of LB1 (62–64). Because the LB1 expression level also decreases when HGPS patient cells become prematurely senescent (65) and PG expression levels increase with age in aortas from G609G/+ mice (33), spontaneous NE rupture might be induced by the HGPS mutation premature senescence and rapid cellular aging. Interestingly, previous studies indicate that chromatin is soft, whereas the NL is stable and stiff in G609G/+ nuclei compared with WT nuclei (51, 66, 67). These results suggest that the structural abnormalities induced by the HGPS mutation could result in spontaneous NE rupture.

Instead of spontaneous NE rupture, we find that the nuclear reentry of NLS-sfCherry is significantly slow in G609G/+ nuclei compared with WT nuclei immediately after the induction of forced NE rupture by single-cell compression. The difference between WT and G609G/+ cells could be undetectable if NE rupture is induced by laser microirradiation because the damage of the NE is too severe to repair the rupture sites compared with single-cell compression (36, 38). For localizing LA/C and PG to the rupture sites, laser microirradiation is used because we can control the timing and location of NE rupture events. The LA expression level in G609G/+ cells is reduced compared with WT cells due to the efficient alternative splicing from the mutant allele of the *Lmna* gene (41, 68). This could keep the nucleoplasmic pool of LA small, resulting in decelerating the localization kinetics at the rupture sites. On the other hand, the LC expression level could be increased in G609G/+ cells compared with WT cells for some reason (40, 41). However, the diffusion of the fast component is significantly slow in the nucleoplasm of G609G/+ nuclei compared with WT nuclei, as our results indicate. This could be explained if there is the possibility that LC is increased in the NL and decreased in the nucleoplasm because of the strong association with the NL by PG expression. To support this idea, LC has a significantly slow turnover in the NL of HGPS patient nuclei compared with normal nuclei (51, 69). These results suggest the possibility that the changes in expression and distribution of LA/C by PG expression might impair the repair of NE rupture.

We find that when the farnesylation at the CaaX motif of pre-LA and pre-PG is inhibited by FTI treatment, they are increased and become diffusible in the nucleoplasm to accelerate the localization kinetics to the rupture sites. These results indicate that the farnesylation process limits the localization to the rupture sites regardless of the second cleavage of pre-LA. Our previous study indicates that the phosphorylation of LA at Ser 22 accelerates the localization kinetics at the rupture sites (38). Interestingly, other studies indicate that PG is not or less phosphorylated at Ser 22, which is prevented by the inhibition of farnesylation (40, 70). Therefore, the acceleration of PG localization to the rupture sites by the inhibition of farnesylation could be mediated through the phosphorylation in part. Furthermore, Zmpste24 KD blocks the second cleavage, leading to the complete inhibition of pre-LA localization to the rupture sites, and the effects of Zmpste24 KD on the NP/NL ratios and the localization kinetics are rescued by FTI treatment. These results strongly suggest that pre-LA is mostly farnesylated in Zmpste24-KD cells and that the nucleoplasmic pool is little or none to localize to the rupture sites within 60–70 min.

Apart from the farnesylation at the CaaX motif and the inhibition of the second cleavage of pre-LA, we narrowed down two functional segments for slowing the localization to the rupture sites, LACS1 and LACS2, within the LA tail region which have been overlooked for decades because these segments are within the intrinsically disordered region. Because the truncation of LACS1 significantly increases the NP/NL ratios, LA appears to be retained in nuclear structure through LACS1 compared with LC, resulting in slow localization. LACS1 may contribute to the interaction of LA with the NL and/or other nuclear compartments. On the other hand, LACS2 could assist the LACS1 function in inhibiting LA localization to the rupture sites without enhancing the retention of LA in the NL. Because the truncation of the residues 599–625 between LACS1 and LACS2 in human increases the NP/NL ratios, a segment within these amino acids is likely to retain its small pool in the nucleoplasm. Whereas the residues 411–553 including the NLS and the Ig-fold domain interact with dsDNAs (71), the residues 506–638 that contain LACSs bind histones (72, 73), suggesting that LA is partially tethered to chromatin through LACSs to slow the localization to the rupture sites.

We demonstrate that the abundance of diffusible LA in the nucleoplasm is affected by the farnesylation at the CaaX motif, the second cleavage, and the retention in nuclear structure through LACSs. On the other hand, LC is free from these rate-limiting factors. Conversely, LA/C is involved in repairing the NE. LA/C KD delays the nuclear reentry of NLS fused to GFP immediately after the induction of NE rupture (24, 25), and LA/C KO reduces the nuclear localization and accumulation of BAF at the rupture sites (38). These results strongly suggest that the abundance of diffusible LC in the nucleoplasm determines the rate of NE repair at the very early stage, whereas LA does not start promoting prolonged recovery from NE rupture until much later. Our findings of the delay in retrieving NLS-sfCherry from the cytoplasm and the slow localization kinetics of LA, LC, and PG at the rupture sites in G609G/+ nuclei implicate the pathological significance of the defective NE repair in HGPS. The therapeutic effect of FTI treatment appears to rescue the repair defect by targeting the farnesylation of PG. Therefore, the rescuing effect of FTI treatment on the defective NE repair needs to be validated using the mouse model of HGPS in future studies.

## Materials and methods

See SI Appendix, SI Materials and methods for plasmid construction.

### Cell culture

MEFs isolated from WT and heterozygous c.1827C > T mutant (*Lmna*^G609G/+^, G609G/+) embryos at embryonic day 13.5 were generously gifted by Carlos López-Otín (Universidad de Oviedo) (41, 42). Primary MEFs were cultured in modified DMEM (high glucose; Nacalai Tesque) containing 10% FBS (qualified; Thermo Fisher), 4 mM L-glutamine, 100 U/mL penicillin and 100 μg/mL streptomycin (Sigma-Aldrich), and 20 mM HEPES (Nacalai Tesque) at 37 °C in a humidified multigas incubator with 3% O_2_/5% CO_2_ (MCO-5 M, Sanyo/Panasonic Healthcare). Cells were immortalized by transduction with pBABE-puro largeTcDNA (Addgene plasmid # 14088; a gift from William Hahn (74)) as previously described (13). After selection with 2 μg/mL puromycin (InvivoGen) for 4 days, immortalized WT and G609G/+ cells were cultured in growth medium without HEPES at 37 °C in a humidified chamber with 5% CO_2_.

### Lentiviral transduction

For lentivirus-mediated stable introduction of NLS^SV40^-sfCherry-NLS^Myc^ (denoted NLS-sfCherry throughout manuscript) and KD of BAF and Zmpste24, we followed the methods that were previously described (38). NLS-sfCherry-expressing cells were selected by incubation in medium containing 3 µg/mL blasticidin S or 200 µg/mL hygromycin B Gold (InvivoGen) for 4 days. shRNAs-expressing cells were selected with 3 µg/mL blasticidin S for 3 days.

### Indirect immunofluorescence and microscopy

For indirect immunofluorescence staining, we followed the methods that were previously described (38). Primary antibodies used for immunofluorescence were mouse monoclonal anti-LA (1:250; 4A58, Santa Cruz), rat monoclonal anti-pre-LA (1:1,000; 7G11; EMD Millipore (55)), mouse monoclonal anti-mature-LA (1:8,000; 4A4-A4, Invitrogen (75)), rabbit polyclonal anti-progerin (1:1,000; RaPG, generated by the Nourshargh laboratory using a peptide immunogen of murine progerin and standard immunization procedures (68, 76, 77)), mouse monoclonal anti-LB1 (1:1,000; B-10; Santa Cruz), and rabbit monoclonal anti-BANF1/BAF (1:200; EPR7668; Abcam). The secondary antibodies used were Alexa Fluor 488-donkey anti-mouse immunoglobulin G (IgG), Alexa Fluor 488-donkey anti-rabbit IgG (1:2,000; A21202 and 1:2,000; A21206, respectively, Thermo Fisher), Cy5-donkey anti-mouse IgG, and Cy5-goat anti-rat IgG (1:2,000; 715–175–151 and 1:2,000; 112-175-167, respectively, Jackson ImmunoResearch).

Exceptionally for Fig. S1A, cells were fixed with 4% PFA for 15 min, followed by permeabilization using 0.1% Triton X-100 in PBS for 10 min and then blocking with Blocking One-P for 30 min. After incubation with primary antibodies, the RFP-Booster Alexa Fluor 568 (alpaca monoclonal anti-RFP Nanobody; 1:1,000; rb2AF568, ChromoTek) was used with second antibodies to amplify the signal of NLS-sfCherry.

Confocal fluorescence images of single confocal sections were obtained using a confocal microscope (FV1000, Olympus) with a 60× PlanApo N (NA 1.4) oil lens operated by built-in software FLUOVIEW ver. 4.2 (3.0% 405-nm laser transmission; 3.1–11.1% 488-nm laser transmission; 5.0% 543-nm laser transmission; 1.0–4.0% 633-nm laser transmission; 2 μs/pixel; 512 × 512 pixels; pinhole 100 μm). CellProfiler ver. 3.1.9 (Broad Institute) with the NP_NL_intensity_fixed pipeline (uploaded on GitHub; http://github.com/Kimura-Lab/Kono-et-al.-2023) was used for measuring mean intensity of lamins in the NP and the NL. The nuclear region was recognized by staining DNA with Hoechst 33342. Fiji software (ImageJ) ver. 1.53v (National Institute of Health) was used for measuring max intensities in the region of interest from nucleoplasm and cytoplasm after Gaussian filtering with *σ* = 2.0 and background subtraction.

### Induction of mechanical NE rupture by single-cell compression

The culture medium was replaced with Leibovitz's L-15 medium (no phenol red; Thermo Fisher) containing 10% FBS, 2 mM L-glutamine, 100 U/mL penicillin, and 100 μg/mL streptomycin. JPK NanoWizard 4 BioAFM (Bruker) incorporated into an inverted fluorescence microscope (Eclipse Ti2, Nikon) with EM-CCD camera (iXon Ultra 888, Andor) and light source (X-Cite XYLIS, Excelitas) was used for single-cell compression. A cantilever (TL-CONT, Nanosensors; spring constant ∼0.2 N/m) with a 100-μm glass bead (BZ-01, As-One) was used for NE rupture. Cells expressing NLS-sfCherry and cGAS-sfGFP were set onto a heated stage (37 °C), and the first image was obtained before the rupture using NIS-Elements BR (Nikon). Next, the bead on the cantilever was approached on the top of the cell surface, and then, the *z*-axis was forced down for 5 μm without feedback by lowering the motor of the AFM head. After waiting for 10 s, the cantilever was retracted. Within 10 s after the retraction, time-lapse imaging was started with 30 s exposure time every 30 s for 5 min. The mean intensity in the region of interest from nucleoplasm, cytoplasm, and background was measured by Fiji/ImageJ after Gaussian filtering with *σ* = 2.0 over time.

### Live-cell imaging and NE rupture induction by laser microirradiation

For live-cell imaging and laser microirradiation to analyze the accumulation dynamics of sfGFP-DARPin-LA6 and mEmerald-lamins at the sites of NE rupture, we followed the methods that were previously described (38). All live-cell confocal images were acquired using a main scanner (4% 488-nm laser transmission; 30% 543-nm laser transmission; 2 μs/pixel; 512 × 512 pixels; pinhole 100 μm; 6× zoom; ∼10 s/frame). After the first image was acquired, a 2-μm-diameter spot was laser-microirradiated using a second scanner at 100% power of 405-nm laser transmission (40 μs/pixel) for 10 s, whereas the images were acquired with another scanner. CellProfiler with the NP_NL_intensity_live pipeline (uploaded on GitHub; http://github.com/Kimura-Lab/Kono-et-al.-2023) (38) was used for measuring mean intensity of mEmerald-lamins in the NP and the NL.

### Chemicals

As a selective FTI, lonafarnib (SCH66336; Merck) was dissolved in dimethylsulfoxide (DMSO; Nacalai Tesque) and added to the cells with one daily dose of 3.2 μM for 2 days. For controls in all experiments, an equal volume of vehicle (0.1% DMSO) was added.

### Immunoblotting

For immunoblotting, we followed the methods that were previously described (38). Primary antibodies used for immunoblotting were mouse monoclonal anti-LA/C (1:5,000–20,000; 3A6-4C11, eBioscience), rat monoclonal anti-pre-LA (1:1,000; 7G11), rabbit polyclonal anti-PG (1:1,000; RaPG, Nourshargh lab), rabbit monoclonal anti-BANF1/BAF (1:500; EPR7668, Abcam), anti-β-actin (1:2,000; AC-15, Santa Cruz), and anti-GAPDH (1:5,000; 6C5, Santa Cruz). The secondary antibodies used were anti-mouse IgG HRP-linked whole Ab sheep (1:10,000; NA931, Amersham), anti-rabbit IgG HRP-linked F(ab’)_2_ fragment donkey (1:10,000; NA9340, Amersham), and HRP-donkey anti-rat IgG (H + L) (1:10,000; 712-035-150, Jackson ImmunoResearch). All images were processed in Photoshop ver. 24 (Adobe) for cropping and brightness/contrast adjustment.

### Rosa26-targeted gene knock-in

The pLmna-Em-pre-LA-R26Neo, pLmna-Em-PG-R26Neo, and pLmna-Em-LC-R26Neo were linearized by the *Kpn* I (Takara) and purified by phenol/chloroform extraction and ethanol precipitation. Cells were transfected with appropriate plasmids using Lipofectamine 3000 (Thermo Fisher) by reverse transfection as previously described (38). Two days after the transfection, cells were selected by incubation in medium containing 400 µg/mL G-418 disulfate aqueous solution (Nacalai Tesque) for 7 days.

### Fluorescence correlation spectroscopy

FCS measurements were all performed at 25 °C with an LSM780 confocal microscope (Carl Zeiss) as previously described (15, 47, 78). FCS setups using the LSM780 microscope consisted of a continuous-wave Ar^+^ laser (25 mW), a water-immersion objective (C-Apochromat, ×40/1.2 NA; Carl Zeiss), and a GaAsP multichannel spectral detector (Quasar; Carl Zeiss). Fluorescent protein of mEmerald was excited with the 488-nm laser line with a minimal laser power (0.5%) to allow an optimal signal-to-noise ratio. Diameter of pinhole was adjusted to 34 μm (1 Airy unit) for the 488-nm laser. Emission fluorescence was detected at 500–550 nm in the green channel. The structure parameter and detection volume were calibrated using Rhodamine-6G (Sigma-Aldrich) solution with diffusion coefficient (280 μm^2^/s; 25 °C in water) before experiments with cells. For FCS measurement, three positions were randomly selected inside the nucleoplasm, and molecular diffusion was measured for 10 s five times. The first round of the five repetitions was carried out to photobleach immobile fraction, and then, the four repetitions were used for further analysis. FCS data were analyzed with analytical software installed in the ZEN 2012 SP5 FP1 (black) acquisition software (Carl Zeiss) (47, 78). Briefly, all measured fluorescence auto-correlation functions (FAFs) from live cells were globally fitted by the ConfoCor3 software installed on LSM780 system using fitting models considering three-dimensional free diffusion with a Gaussian laser profile, including a triplet component to estimate the diffusion coefficient.

### Statistical analyses

Unless mentioned otherwise, all plots showed mean values ± SEM (error bars). Fisher's exact test was performed to analyze the categorical data using EZR on R commander ver. 1.61 (programmed by Yoshinobu Kanda) (79). The mixed-effects model fit by restricted maximum likelihood was performed to analyze the interaction between group and time using the *lme4* (80) and the *lmerTest* (81) packages in EZR, where cell's id was set as a random effect, while group and time as fixed effects. The interaction between group and time (group × time) was also set as a fixed effect. The Welch's t test or the Welch's one-way ANOVA followed by the Games–Howell post-hoc test was performed to single or multiple comparison, respectively. Statistic codes for R ver. 4.2.2 and a modified R commander for EZR 1.61 are uploaded on GitHub (http://github.com/Kimura-Lab/Kono-et-al.-2023). Significance was determined if *P* < 0.05.

## Supplementary Material

pgae527_Supplementary_Data

## Data Availability

All the data supporting the findings of this study are included in the main text and/or SI Appendix. All analysis R codes and CellProfiler pipelines are freely available on our GitHub page: http://github.com/Kimura-Lab.

## Preprints

This manuscript was posted on a preprint: http://doi.org/10.1101/2023.09.02.555826.
