# Supplementary material for: Roles of the lamin A-specific tail region in the localization to sites of nuclear envelope rupture
Source: PNAS Nexus. 2024 Nov 21;3(12):pgae527. doi: 10.1093/pnasnexus/pgae527 (PMC11645434; doi:10.1093/pnasnexus/pgae527)
Supplement: pgae527_Supplementary_Data [file pgae527_supplementary_data.zip › PNASNEXUS-PNASNEXUS-2024-00475RRRR-s01.pdf]

Roles of the Lamin A-specific Tail Region in the Localization to Sites of Nuclear Envelope Rupture

Yohei Kono, Chan-Gi Pack, Takehiko Ichikawa, Arata Komatsubara, Stephen A. Adam, Keisuke Miyazawa, Loïc Rolas, Sussan Nourshargh, Ohad Medalia, Robert D. Goldman, Takeshi Fukuma, Hiroshi Kimura, Takeshi Shimi

Takeshi Shimi
Email: t-shimi@staff.kanazawa-u.ac.jp

This PDF file includes:

SI Materials and methods
Tables S1 and S2
Figures S1 to S14
Legends for Movies S1 and S2
SI References

Other supplementary materials for this manuscript include the following:

Movies S1 and S2

SI Materials and methods

Plasmid construction

Two different NLSs derived from SV40 large T antigen (NLS^{SV40}; PKKKRKV) and c-Myc (NLS^{Myc}; PAAKRVKLD) from pCDH-NLS^{SV40}-sfCherry-NLS^{Myc}-Blast (1) were ligated to pCDH-CMV-MCS-EF1-Hygro (plasmid #CD515B-1, System Biosciences) using the Ligation-Convenience Kit (Nippon Gene) and designated as pCDH-NLS^{SV40}-sfCherry-NLS^{Myc}-Hygro. The mouse cGAS cDNA, generated by RT-PCR with cDNA library prepared from A9 fibroblasts (JCRB0211, JCRB Cell Bank) (2) using the PrimeSTAR HS DNA Polymerase (Takara), was fused to the C-terminus of sfGFP using the In-Fusion HD Cloning Kit (Clontech). The resulting plasmid was designated as pmcGAS-sfGFP. Primers used in this study are listed in Table S2. A9 cells were generous gift from Mitsuo Oshimura (Tottori University) (3), and total RNA was extracted using TRIzol reagent (Thermo Fisher) (4). The catalytically inactive cGAS mutant E211A/D213A in the DNA-stimulated nucleotidyl transferase domain (5, 6), was generated by PCR mutagenesis using PrimeSTAR HS. The annealed oligonucleotides to express shRNAs (BAF#1, TRCN0000124958; BAF#2, TRCN0000124955; Zmpste24, TRCN0000366641; Table S2, Broad Institute) were ligated into pLKO.3-blast (1) using the Ligation-Convenience Kit. The pX459-sfCherry CRISPR-Cas9 vector were generated from pSpCas9(BB)-2A-Puro (pX459) V2.0 (Addgene plasmid # 62988 ; http://n2t.net/addgene:62988 ; RRID:Addgene_62988; a gift from Feng Zhang (7)). The annealed oligonucleotides to express single-guide RNA (sgRNA) against the *Rosa26* target sequence (8) was ligated into pX459-sfCherry using the Ligation-Convenience Kit and designated as pX459-sfCherry-sgRosa26-1.

We cloned a 1.6 kb genomic region upstream from start codon of mouse *Lmna* as the endogenous promoter (-1407 to +249 bp from transcription start site) using PrimeSTAR HS. Mouse genomic DNA was extracted from cells by Proteinase K (Fungal; Invitrogen) digestion according to the standard protocol and used for a PCR template. The CMV promoter of mEmerald-C1 (Addgene plasmid # 53975 ; http://n2t.net/addgene:53975 ; RRID:Addgene_53975; a gift from Michael Davidson) was replaced by the *Lmna* promoter using the In-Fusion and designated as pLmna-mEmerald-C1. The mouse pre-LA cDNA was amplified from pPyCAG-LA-IP (9) using the KOD One PCR Master Mix -Blue- (Toyobo), cloned into pLmna-mEmerald-C1 using the In-Fusion, and designated as pLmna-Em-LA. The mouse PG cDNA was generated by PrimeSTAR Max DNA Polymerase (Takara) to skip 150 bp, closed by In-Fusion HD, and designated as pLmna-Em-PG. The amino acid truncation mutants were generated by PCR mutagenesis using PrimeSTAR Max or PrimeSTAR HS. The LACS1 deletion mutant (Δ26) was generated using PrimeSTAR Max to skip 78 bp, and closed by In-Fusion HD. The pLmna-Em-LA and pLmna-Em-PG expression cassettes were amplified by the KOD One, fused with bGHpA amplified from LSL-Cas9-Rosa26TV (Addgene plasmid # 61408 ; http://n2t.net/addgene:61408 ; RRID:Addgene_61408; a gift from Feng Zhang (10)) into *Pac* I/*Sma* I-digested LSL-Cas9-Rosa26TV by In-Fusion HD, and designated as pLmna-Em-LA-R26Neo, pLmna-Em-PG-R26Neo and pLmna-Em-PG-CSM-R26Neo. The mouse LC cDNA was also generated by KOD One PCR and In-Fusion HD, and designated as pLmna-Em-LC-R26Neo.

Table S1. Phenotype characterization of WT and G609G/+ MEFs. NLS-sfCherry expressing WT and G609G/+ cells were stained with Hoechst 33342 for DNA and RFP-Booster Alexa Fluor 568 to enhance the signal of NLS-sfCherry to detect spontaneous NE rupture. Immunostaining with anti-LB1 was also used as NE rupture markers. See Fig. S1 A for the representative single confocal images. *P* values, Fisher's exact tests between WT and G609G/+.

	WT (n = 120 cells, 129 nuclei)	G609G/+ (n = 118 cells, 131 nuclei)	P value
DNA			0.465
normal	122 (94.6%)	120 (91.6%)	
misshapen	7 (5.4%)	11 (8.4%)	
NLS-sfCherry			1
nuclear-localized	118 (98.3%)	117 (99.2%)	
ruptured	2 (1.7%)	1 (0.8%)	
α-LB1			0.212
normal distributed	125 (96.9%)	130 (99.2%)	
mis-localized	4 (3.1%)	1 (0.8%)	

Table S2. Oligonucleotides used in this study. See SI Materials and methods. Oligonucleotides were synthesized by Sigma-Aldrich or Integrated DNA Technologies (IDT).

No.	Name	Sequence (5' to 3')
1	if_m-Cgas_Fw	CGCTAGCGCTACCGGTATGGAAGATCCGCGTAGAAGGACG
2	if_m-Cgas_Rv	GCTCACCATGGTGGCAAGCTTGTCAAAAATTGGAAACCCATTATTTC
3	mCgas-E211A_D213A_Fw	GATTTCTGCTCCTAATGCATTTGCTGTTATGTTTAAACTG
4	mCgas-E211A_D213A_Rv	CAGTTTAAACATAACAGCAAATGCATTAGGAGCAGAAATC
5	shBanf1#1-TRCN0000124958_s	CCGGTGGCCAGTTTCTGGTGCTAAACTCGAGTTTAGCACCAGAAACTGGCCATTTTTG
6	shBanf1#1-TRCN0000124958_as	AATTCAAAAATGGCCAGTTTCTGGTGCTAAACTCGAGTTTAGCACCAGAAACTGGCCA
7	shBanf1#2-TRCN0000124955_s	CCGGGACAAGGCTTATGTGGTCCTTCTCGAGAAGGACCACATAAGCCTTGTCTTTTTG
8	shBanf1#2-TRCN0000124955_as	AATTCAAAAAGACAAGGCTTATGTGGTCCTTCTCGAGAAGGACCACATAAGCCTTGTC
9	shSte24#1-TRCN0000366641_s	CCGGCTGGATGCTCTTCCGGTTATTCTCGAGAATAACCGGAAGAGCATCCAGTTTTTG
10	shSte24#1-TRCN0000366641_as	AATTCAAAAACTGGATGCTCTTCCGGTTATTCTCGAGAATAACCGGAAGAGCATCCAG
11	pX459V2-Fw	GAATTCTAACTAGAGCTCGCTGATCAGCCTCG
12	pX459V2-Rv	TGGGCCAGGATTCTCCTCGACGTCAC
13	if_mCherry_Fw	GAGAATCCTGGCCCAATGGTGAGCAAGGGCGAGGAGG
14	if_mCherry_Rv	CTCTAGTTAGAATTCTCACTTGTACAGCTCGTCCATGCCGCCGG
15	sgRosa26-1_s	CACCGACTCCAGTCTTTCTAGAAGA
16	sgRosa26-1_as	AAACTCTTCTAGAAAGACTGGAGTC
17	if_Lmna promoter_Fw	ATGCATTAGTTATTAATTGCCTCGAAAAACCAAAAAAAG
18	if_Lmna promoter_Rv	GACCGGTAGCGCTAGCCCGCTGTGACGGGGTCTCCATG
19	if_mLA_Fw	ATGGAGACCCCGTCACAGCGG
20	if_mLA_Rv	AACCGGTAGCGCTAGCCATGATGCTGCAGTTCTGGG
21	mLA-to-PG_Fw	CAGAGCTCCCAGAACTGCAGC
22	mLA-to-PG_Rv	TCTGGGAGCTCTGGGCTCCCGCTCCACCG
23	mLA-L648X_Fw (mature)	CACCCGCTCCTACTAGCTGGGCAACTCC
24	mLA-L648X_Rv (mature)	GGAGTTGCCCAGCTAGTAGGAGCGGGTG
25	mLA-T644X_Fw	GACAACCTAGTCTAACGCTCCTACCTC
26	mLA-T644X_Rv	GAGGTAGGAGCGTTAGACTAGGTTGTC
27	mLA-F627X_Fw	GTCACTCGAAGCTGACGCAGTGTGGGG
28	mLA-F627X_Rv	CCCCACACTGCGTCAGCTTCGAGTGAC
29	mLA-S617X_Fw	CCTCTGGCTCTTGAGCCTCCAGTGTC
30	mLA-S617X_Rv	GACACTGGAGGCTCAAGAGCCAGAGG
31	mLA-A601X_Fw	GCTGACAAGGCTTGAGGTGGAGCGGGAG
32	mLA-A601X_Rv	CTCCCGCTCCACCTCAAGCCTTGTCAGCAG
33	mLA-S575X_Fw	CACTGCAGCGGCTAGGGGGACCCCGC
34	mLA-S575X_Rv	GCGGGGTCCCCCTAGCCGCTGCAGTG
35	if_mLA-601_Fw (Δ26)	CAGCGGCGCCGGTGGAGCGGGAGCCC
36	if_mLA-574_Rv (Δ26)	CCACCGGCGCCGCTGCAGTGGGAACCAC
37	if_Rosa26L-mLA_Fw	GTCTTTCTAGCCTTAATTAATGCCTCGAAAAACCAAAA
38	if_mLA-bGHpA-Rv	GAGGTCGACCAATTGTTACATGATGCTGCAGTTCTG
39	if_mPG-CSM-bGHpA-Rv	GAGGTCGACCAATTGTTACATGCTGCAGTTCTGGGAGCT
40	if_bGHpA_Fw	CAATTGGTCGACCTCGACCTCGACTG
41	if_Rosa26R-bGHpA_Rv	GCGGCCGCCGCGCCCACGCGTTCCCCAGCATGCC
42	if_mLA-to-mLC_Fw	GGCAGCCGCCGCTGAATGCATCAATTGGTCGACCTCGACCTCGACTGT
43	if_mLA-to-mLC_Rv	TCAGCGGCGGCTGCCACTCACACGGTGGTGATGGAGGAGCTCTTCT

Fig. S1

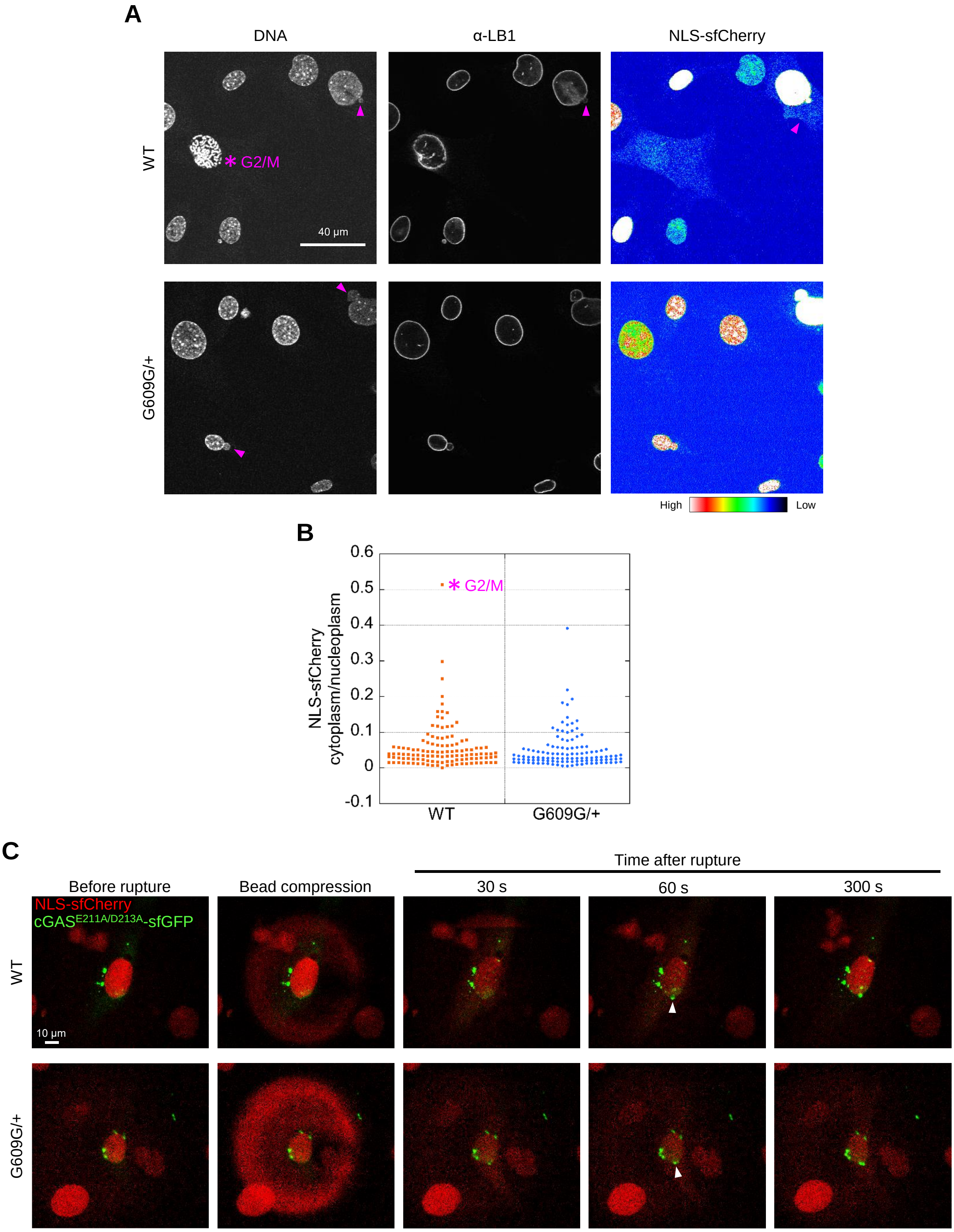
A
DNA
α-LB1
NLS-sfCherry
WT
G609G/+
* G2/M
40 µm
High
Low
B
NLS-sfCherry
cytoplasm/nucleoplasm
0.6
0.5
0.4
0.3
0.2
0.1
0
-0.1
* G2/M
WT
G609G/+
C
Time after rupture
Before rupture
Bead compression
30 s
60 s
300 s
NLS-sfCherry
cGAS^E211A/D213A-sfGFP
WT
G609G/+
10 µm

D

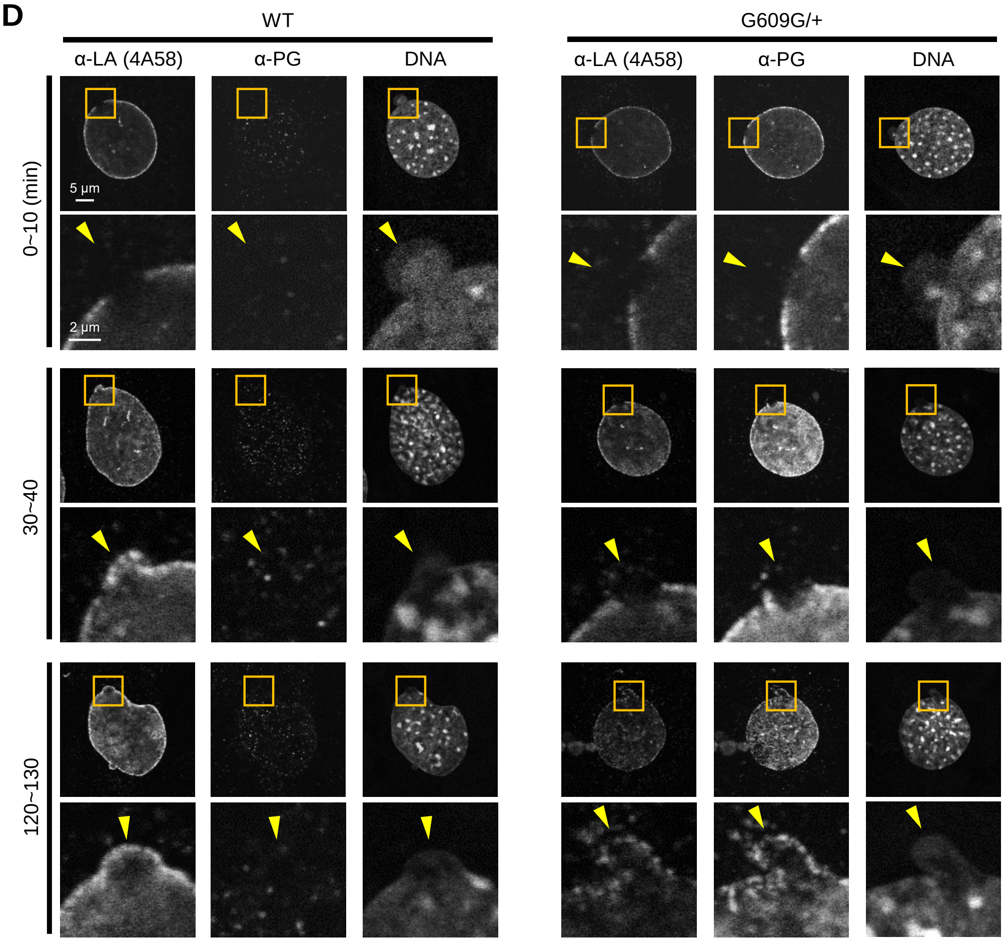
WT
G609G/+
α-LA (4A58)
α-PG
DNA
α-LA (4A58)
α-PG
DNA
0~10 (min)
30~40
120~130
5 μm
2 μm

E

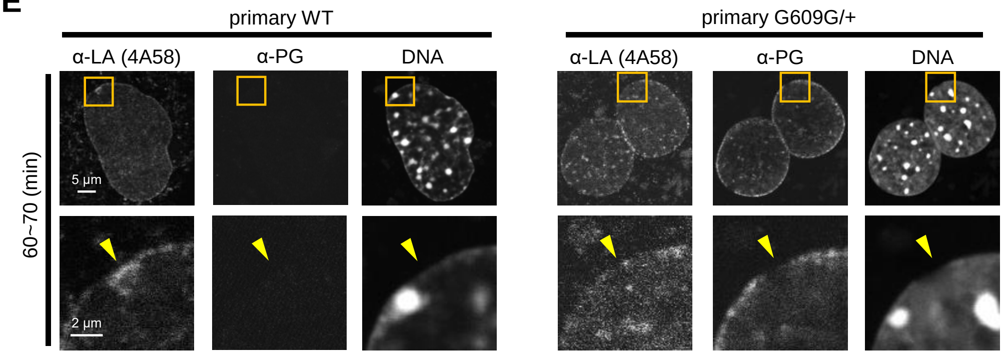
primary WT
primary G609G/+
α-LA (4A58)
α-PG
DNA
α-LA (4A58)
α-PG
DNA
60~70 (min)
5 μm
2 μm

F

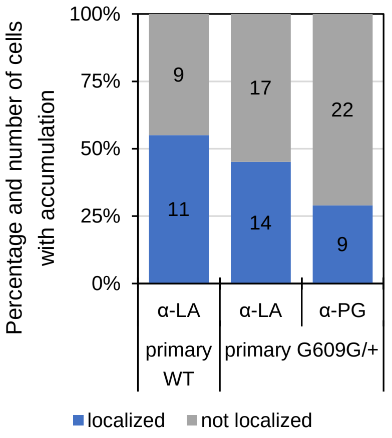
Percentage and number of cells with accumulation
100%
75%
50%
25%
0%
9
11
17
14
22
9
α-LA
α-LA
α-PG
primary WT
primary G609G/+
localized
not localized

G

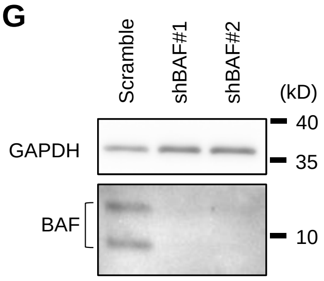
Scramble
shBAF#1
shBAF#2
(kD)
GAPDH
BAF
40
35
10

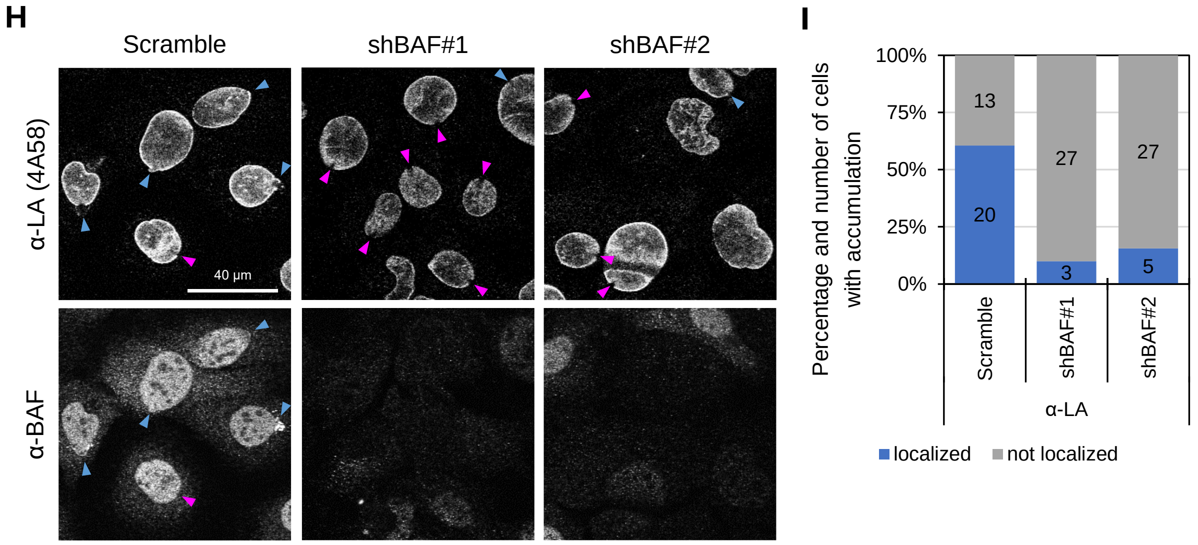

Fig. S1. Phenotype characterization, the localization kinetics of LA and PG at the rupture sites in WT and G609G/+ MEFs, and the localization kinetics of LA at the rupture sites in BAF-KD WT MEFs. The phenotype of heterozygous knock-in G609G/+ expressing NLS-sfCherry was characterized by immunofluorescence. **(A)** Representative confocal images of the cells, which were immunostained with anti-LB1, and the DNA was stained with Hoechst 33342. The fluorescence intensities of NLS-sfCherry are shown with rainbow color. Asterisk indicates a cell with NE breakdown due to the G2-M transition and were excluded from the analysis. Misshapen, NE-ruptured, and LB1-mis-localized nuclei are indicated with magenta arrowheads. **(B)** The cytoplasmic-to-nuclear intensity (C/N) ratios of NLS-sfCherry were measured and plotted. **(C)** Time-lapse images of WT and G609G/+ cells expressing NLS-sfCherry and cGAS$^{E211A/D213A}$-sfGFP were acquired with 30 s intervals for 5 min after the induction of NE rupture by single-cell compression. Bar: 10 μm. **(D and E)** Representative confocal images of the nuclei. Magnified views of the indicated areas by orange boxes are shown (bottom of each row). Bars: 5 μm (top of each row) and 2 μm (bottom of each row). The immortalized MEFs **(D)** were fixed within 0-10 min (top), 30-40 min (middle) and 120-130 min (bottom) and the primary MEFs **(E)** were fixed within 60-70 min after the induction of NE rupture by laser microirradiation, followed by fixation with 4% PFA/0.1% Triton-X 100. The fixed cells were immunostained with anti-LA (4A58) and anti-PG. The rupture sites are indicated with yellow arrowheads (bottom of each row). **(F)** Percentiles of cells with (blue) and without (gray) the localization of LA and PG at the rupture sites. **(G)** Whole cell lysates from WT cells expressing shRNAs for the control (Scramble) or two shRNAs (shBAF #1 and #2) were probed with anti-BANF1/BAF and anti-GAPDH (as loading control) for immunostaining. Positions of the size standards are shown on the right. **(H and I)** 15 to 19 of the indicated cells were laser-microirradiated within 10 min and incubated for 60 min, followed by fixation with 4% PFA/0.1% Triton-X 100. The fixed cells were immunostained with the anti-LA and anti-BAF. **(H)** Representative confocal images of nuclei with (blue arrowheads) or without (magenta arrowheads) the localization of LA to the rupture sites. **(I)** Percentiles of cells with (blue) and without (gray) the localization of LA. **(A and H)** Bar: 40 μm. **(F and I)** The numbers of analyzed cells from two independent experiments are indicated in the bar charts.

Fig. S2

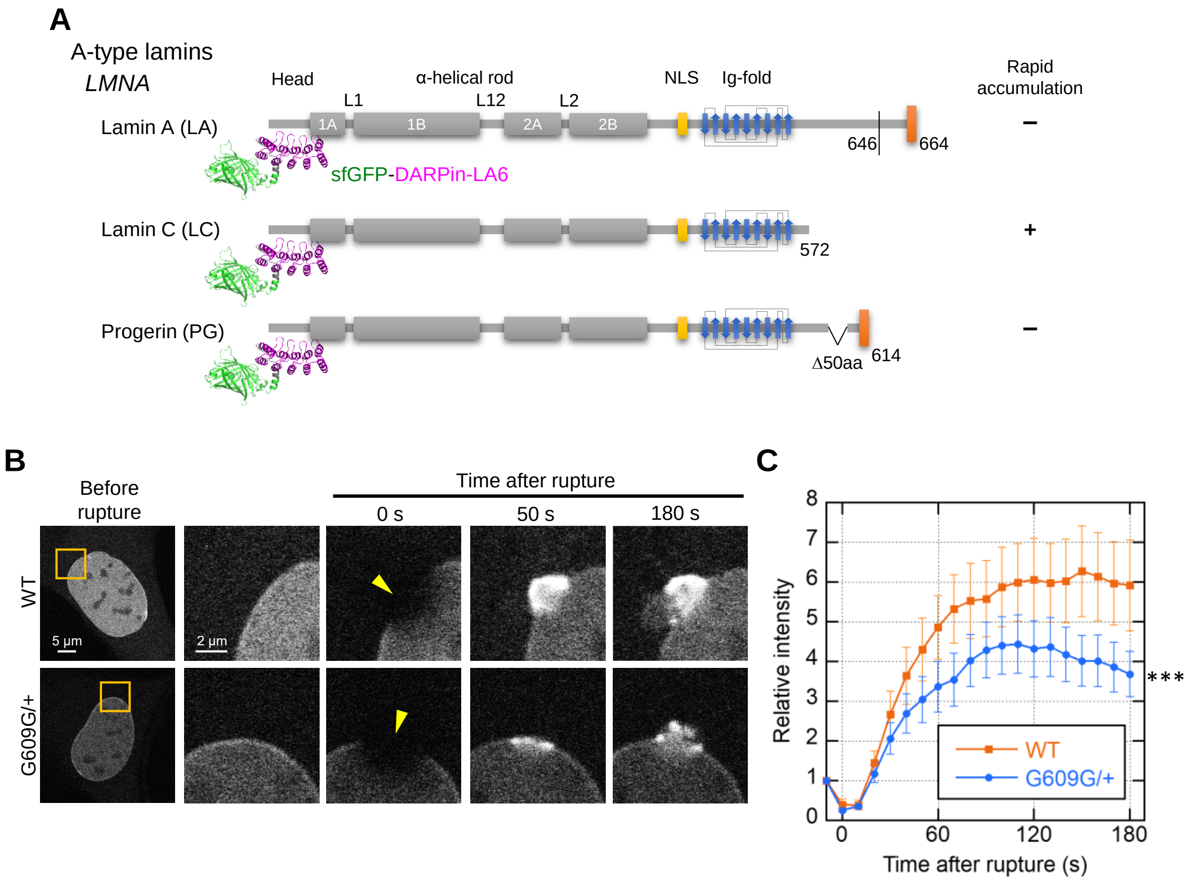

Fig. S2. Accumulation kinetics of LC at the rupture sites in WT and G609G/+ MEFs. (A) Protein architecture of LA, LC and PG with sfGFP-DARPin-LA6. The summary of their accumulation at the rupture sites is indicated on the right (+, accumulated at the rupture site; -, not accumulated). The structural image of sfGFP-DARPin-LA6 was predicted by AlphaFold2 and edited by the PyMOL. **(B and C)** Time-lapse images of sfGFP-DARPin-LA6 expressed in WT and G609G/+ cells were acquired with 10-s intervals for 3 min after the induction of NE rupture by laser-microirradiation, and the relative intensities are plotted in the graph. **(B)** The dynamics of the sfGFP-DARPin-LA6 in response to NE rupture in the cells. Magnified views of the areas indicated by orange boxes are shown (the second to fifth columns). A 2-µm diameter spot at the NE was laser-microirradiated to induce NE rupture (yellow arrowheads). Bars: 5 µm (the first column) and 2 µm (the second to fifth columns). **(C)** The fluorescence intensities at the rupture sites were measured and normalized to the initial intensities (means ± SEM; n = 20 cells from two independent experiments; ***, $P < 0.001$ from WT by a mixed effect model).

Fig. S3

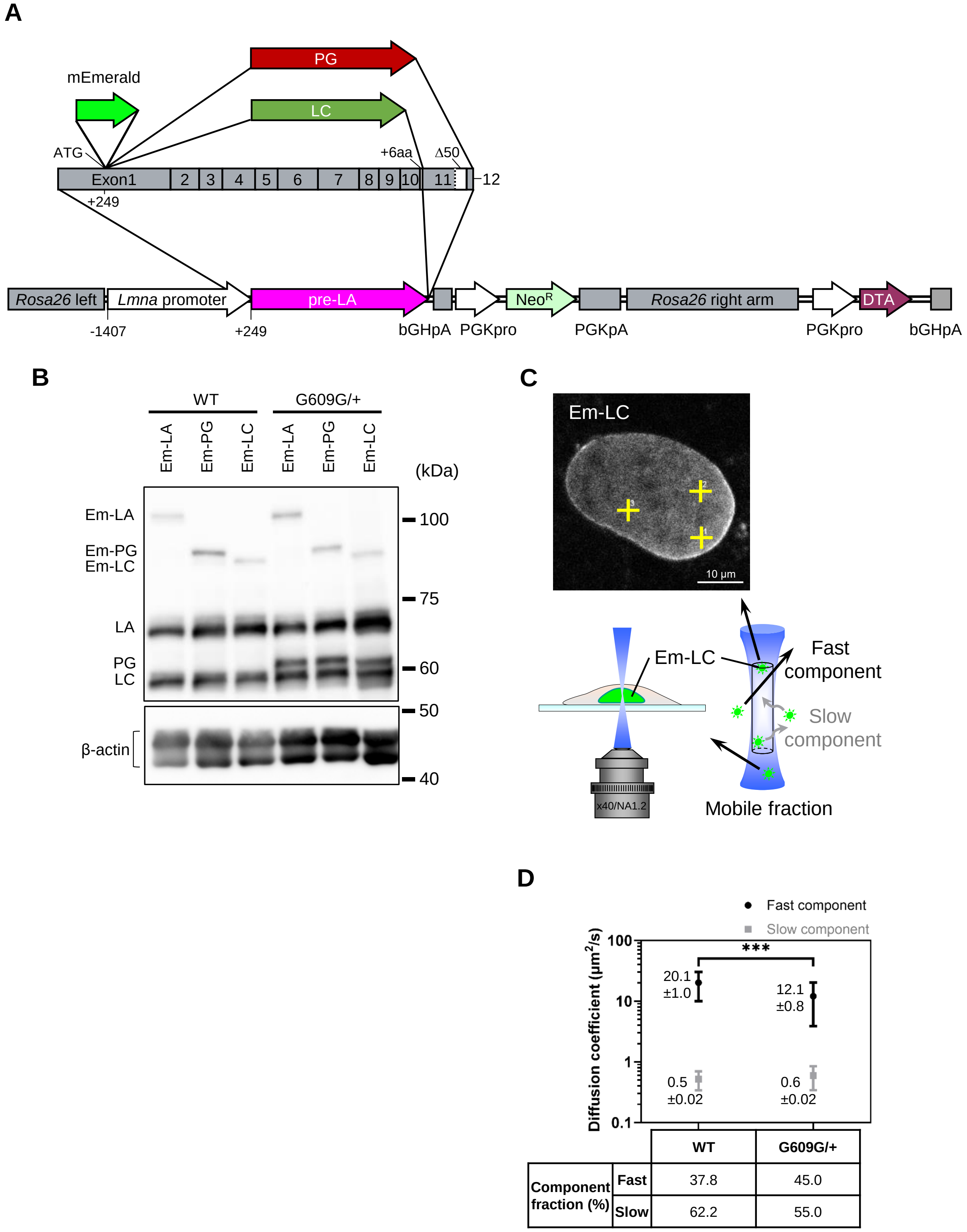

Fig. S3. Establishment of knock-in cells expressing mEmerald-fused LA, PG and LC under the *Lmna* promoter for FCS measurements. The DNA sequences of mEmerald-LA, mEmerald-PG and mEmerald-LC with the endogenous *Lmna* promotor were knocked-in to the *Rosa26* locus in WT and G609G/+ cells. **(A)** The schematic diagram of linearized pLmna-Em-LA-R26Neo, pLmna-Em-PG-R26Neo and pLmna-Em-LC-R26Neo knock-in vectors. **(B)** Whole cell lysates from the knocked-in cells with pLmna-Em-LA-R26Neo, pLmna-Em-PG-R26Neo and pLmna-Em-LC-R26Neo were probed with anti-LA/C (3A6-4C11) and anti-β-actin (as loading control) for immunostaining. Positions of the size standards are shown on the right. **(C and D)** The diffusion

fractions and coefficients of mEmerald-LC (Em-LC) in WT and G609G/+ nuclei were measured by FCS. **(C)** A representative confocal image of a WT nucleus with mEmerald-LC before FCS measurements (top). The yellow crosses indicate the points measured by FCS. Bar: 10 μm. Em-LC molecules move in or out of the confocal volume (white-out cylinder region in blue) at different speeds, as shown in the diagram (bottom). **(D)** Diffusion coefficient of fast component (plotted in black) and slow component (plotted in gray) for Em-LC in the cells. Mean ± SEM are indicated on the left to plots; n = 10 cells from two independent experiments; ***, $P < 0.001$ by a Welch's t-test. Percentiles of these components are indicated at the bottom of the graph.

Fig. S4

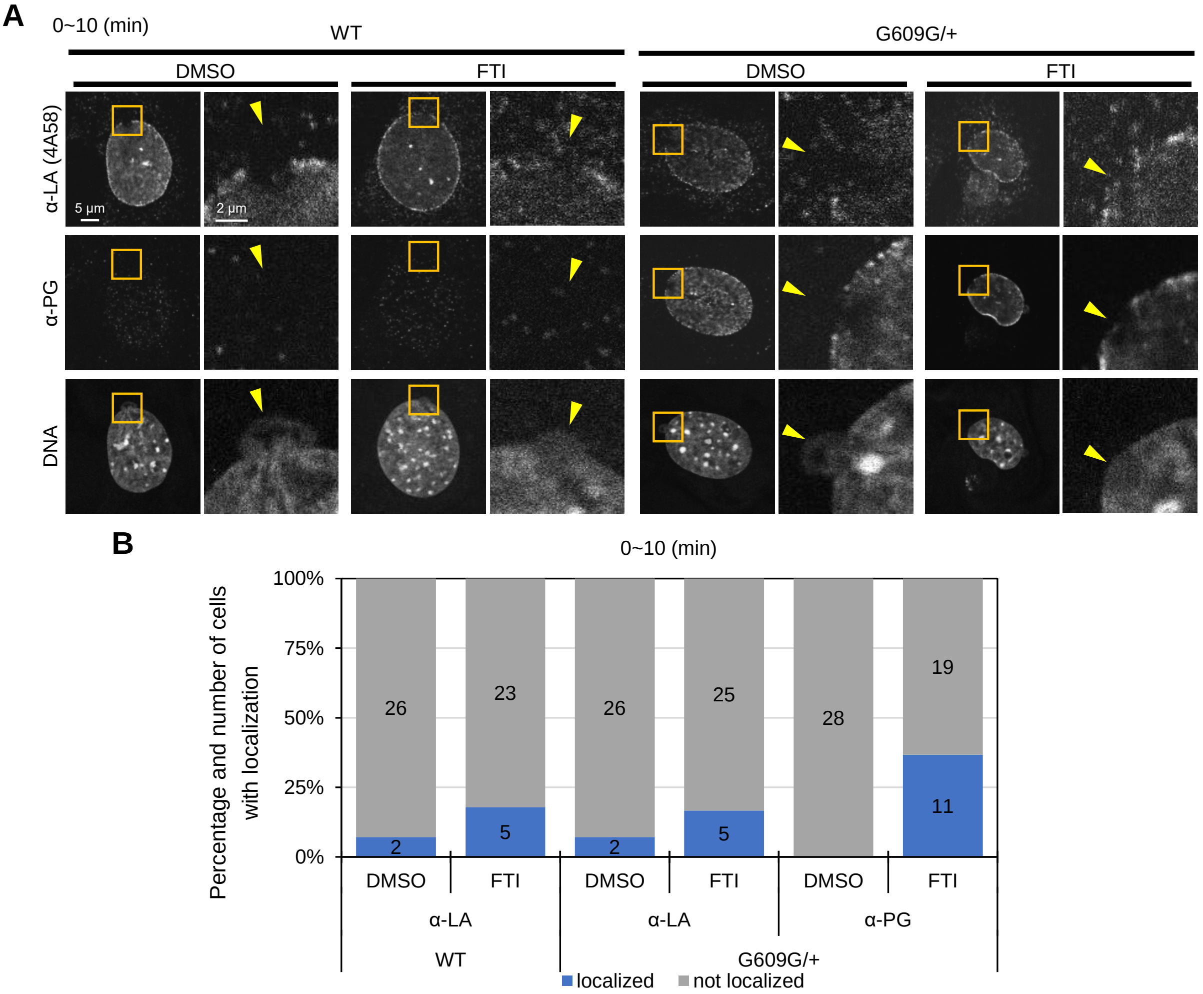

localized	0~5min	1	2	0	0	0	0
	5~10min	1	3	2	5	0	11
not localized		26	23	26	25	28	19

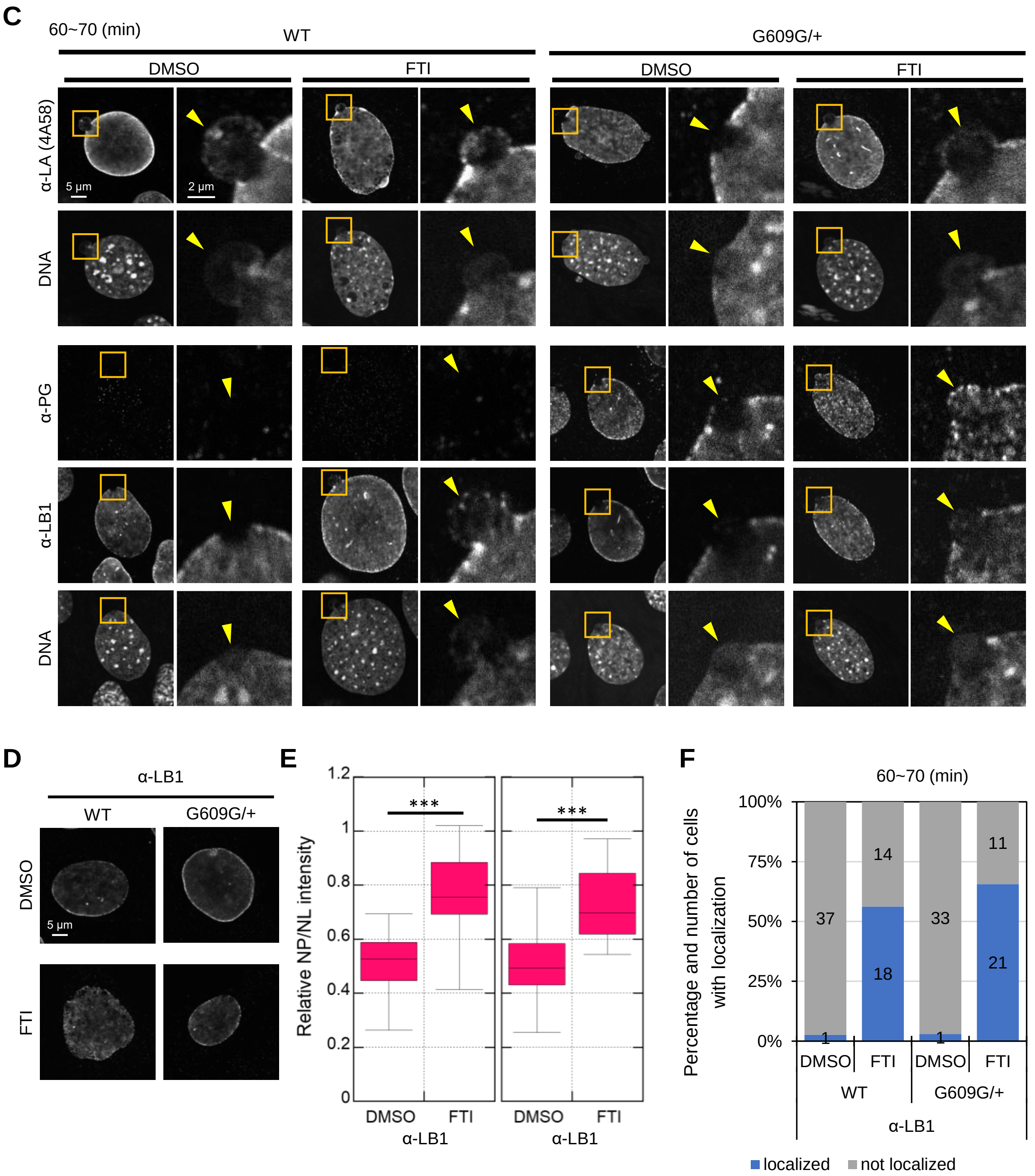

Fig. S4. Localization of LA, PG and LB1 to the rupture sites in DMSO or FTI-treated WT and G609G/+ nuclei. (A and B) 14 to 15 of nuclei were laser-microirradiated within 10 min, followed by fixation with 4% PFA/0.1% Triton-X 100. The fixed cells were immunostained with a combination of the anti-LA and anti-PG. **(A)** Representative confocal images of the nuclei. **(B)** Percentiles of the cells with (blue) and without (gray) the localization of LA and PG at the rupture sites. The number of the cells in which LA and PG were localized to the rupture sites within 5 or 10 min are indicated at the bottom of the graph. **(C)** Representative confocal images of Fig. 2D. **(D and E)** The indicated cells were immunostained with the anti-LB1. **(D)** Representative confocal images of single sections in the middle of the nuclei. Bar: 5 µm. **(E)** Ratios of the average fluorescence intensity in the nucleoplasmic pool relative to that of the NL (NP/NL ratios) of LB1 was measured based on immunofluorescence (n = 20 cells from two independent experiments; ***, $P < 0.001$ by Welch's t-tests). **(F)** Percentiles of the cells with (blue) and without (gray) the localization of LB1 at the rupture sites. **(A and C)** Magnified views of the indicated areas by orange boxes are shown (right of each line). Bars: 5 µm (left of each

line) and 2 μm (right of each line). **(B** and **F)** The numbers of analyzed cells from two independent experiments are indicated in the bar charts.

Fig. S5

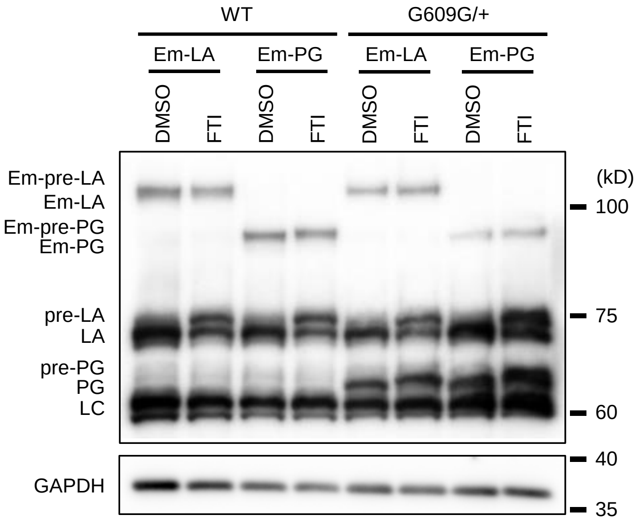

Fig. S5. The immunoblotting of FTI-treated WT and G609G/+ knocked-in MEFs. Whole cell lysates from the knocked-in cells with pLmna-Em-LA-R26Neo (Em-LA) and pLmna-Em-PG-R26Neo (Em-PG) treated with DMSO or FTI were probed with anti-LA/C (3A6-4C11) and anti-GAPDH (as loading control) for immunostaining. Positions of the size standards are shown on the right.

Fig. S6

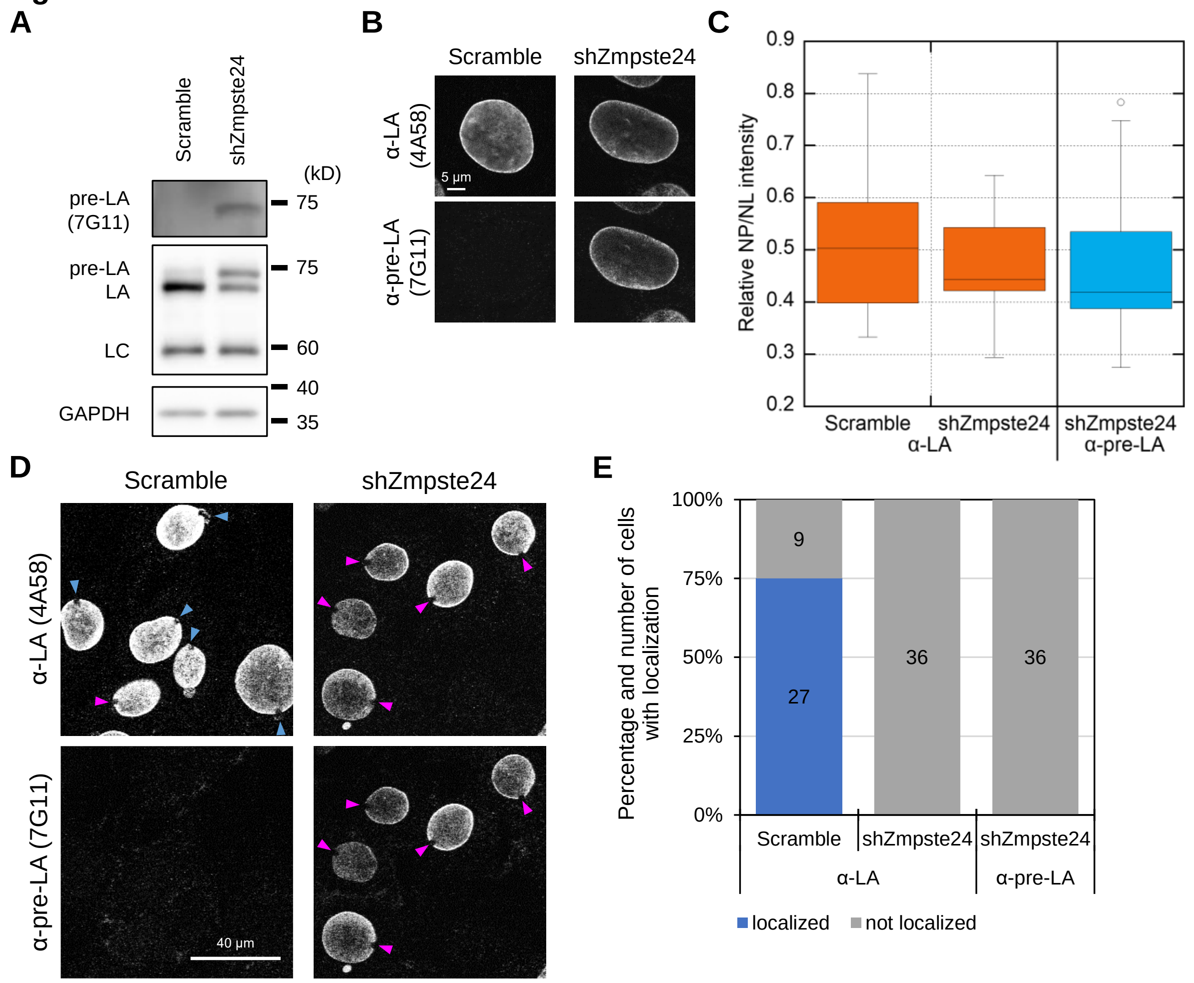

Fig. S6. The localization kinetics at the rupture sites in the control and Zmpste24-KD WT MEFs. (A) Whole cell lysates from WT cells expressing Scramble for the control or shZmpste24 for Zmpste24-KD were probed with anti-pre-LA (7G11), anti-LA/C (3A6-4C11) and anti-GAPDH (as loading control) for immunobloting. Positions of the size standards are shown on the right. **(B** and **C)** The cells were immunostained with anti-LA (4A58) and the pre-LA (7G11). (**B**) Representative confocal images of the nuclei. Bar: 5 µm. **(C)** NP/NL ratios of LA and pre-LA were measured based on immunofluorescence (n = 20 cells from two independent experiments). **(D** and **E)** 18 of nuclei were laser-microirradiated within 10 min and incubated for 60 min, followed by fixation with 4% PFA/0.1% Triton-X 100. The fixed cells were immunostained with the anti-LA and anti-pre-LA. **(D)** Representative confocal images of the nuclei with (blue arrowheads) or without (magenta arrowheads) the localization of LA and pre-LA to the rupture sites. Bar: 40 µm. **(E)** Percentiles of the cells with (blue) and without (gray) the localization of LA and pre-LA. The numbers of analyzed cells from two independent experiments are indicated in the bar charts. **(A and C)** Bars: 5 µm (left of each line) and 2 µm (right of each line).

Fig. S7

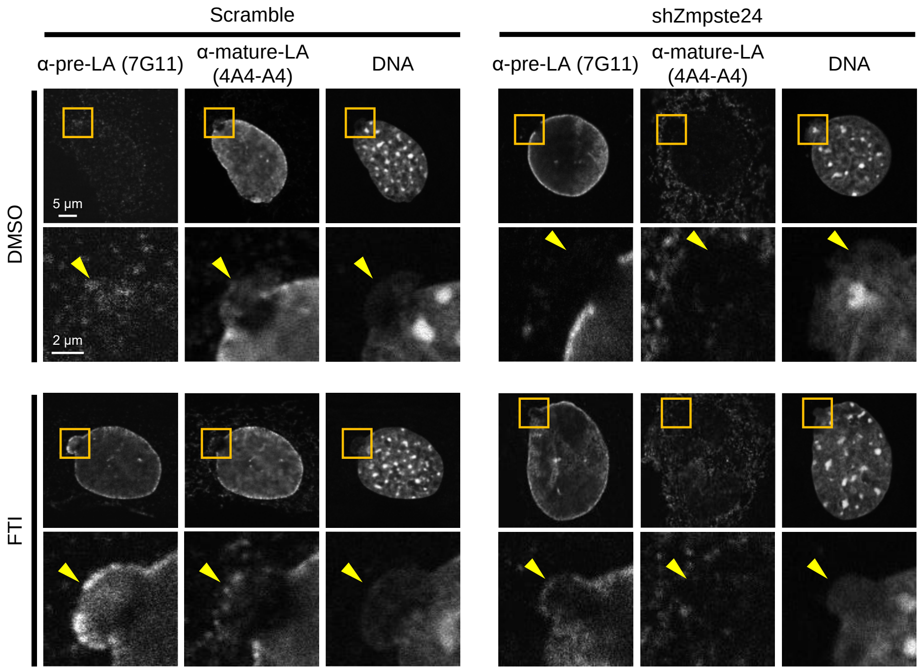

Fig. S7. The localization of pre- and mature-LA to the rupture sites in the control and Zmpste24-KD WT MEFs treated with DMSO or FTI. Representative confocal images of Fig. 4E. Magnified views of the indicated areas by orange boxes are shown (bottom of each row). Bars: 5 μm (top of each row) and 2 μm (bottom of each row).

Fig. S8

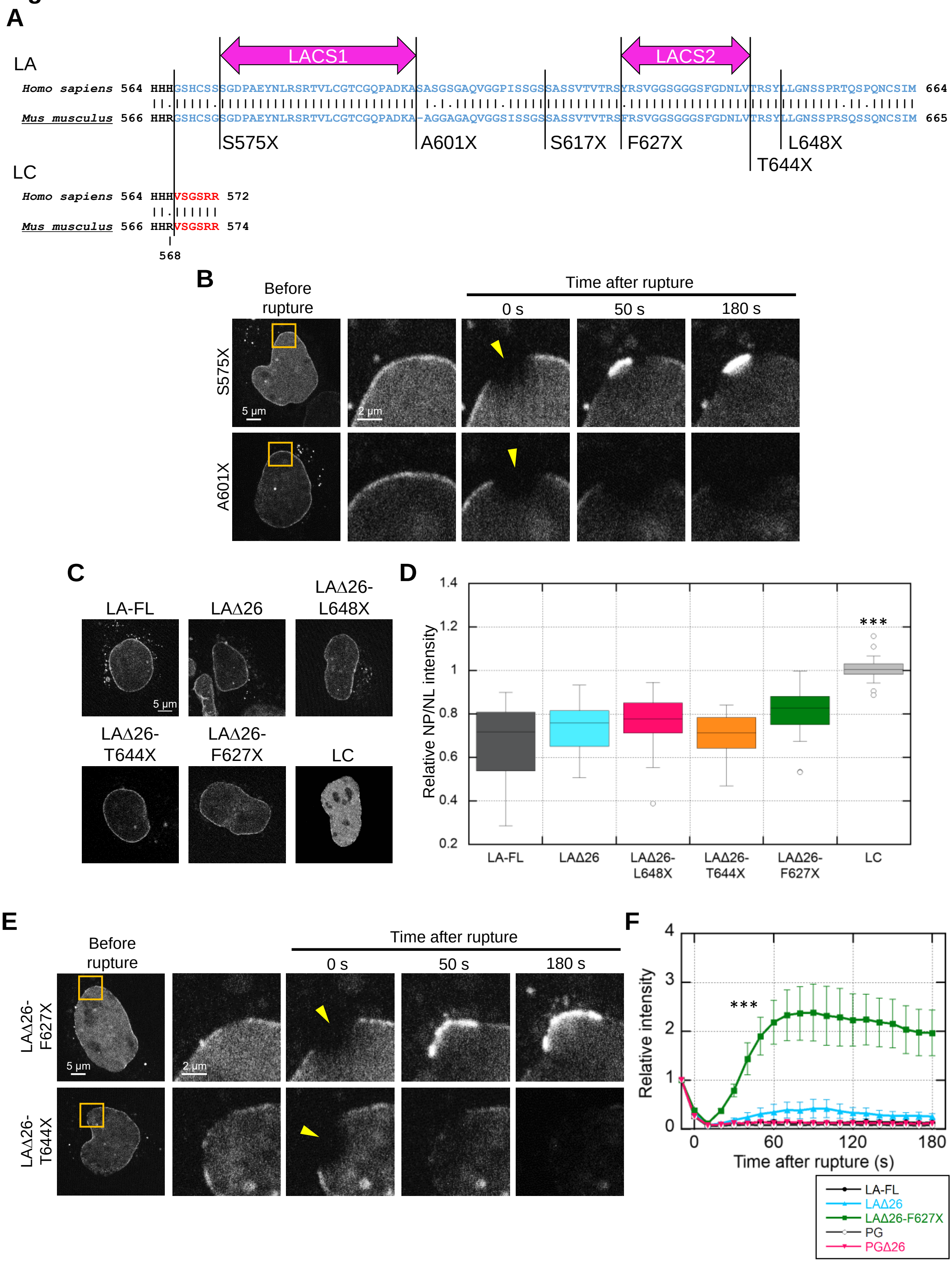

Fig. S8. Accumulation kinetics of LA and PG mutants at the rupture sites in LA/C-KO MEFs. **(A)** Amino acid sequences of LA- (blue) or LC-specific (red) regions from *Homo sapiens* and *Mus musculus*. The amino acid homology is indicated as bars and a gap is introduced between residue A600 and A601 in *Mus musculus* to maximize the homology. **(B)** Dynamics of the mEmerald-LA-S575X and A601X in response to NE rupture in LA/C-KO MEFs. **(C)** Representative images of mEmerald-LA-full-length (FL), LA-FL without LACS1 (LAΔ26), LAΔ26-L648X, LAΔ26-T644X, LAΔ26-F627X, and mEmerald-LC in LA/C-KO MEFs transfected for 2 d. Bar:

5 μm. **(D)** Fluorescence intensities in the NP to the NL ratio of mEmerald-LA mutants and mEmerald-LC, as control, were measured (*n* = 14-20 cells from two independent experiments; ***, $P < 0.001$ by a Games–Howell test). (**C** and **D**) LA-FL and LC are reproduction of Fig. 5B and C. **(E)** Dynamics of the mEmerald-LAΔ26-F627X and LAΔ26-T644X in response to NE rupture in LA/C-KO MEFs. **(F)** Time-lapse images of mEmerald-PGΔ26 were acquired with 10-s intervals for 3 min after the induction of NE rupture by laser-microirradiation, and the relative intensities at the rupture sites are plotted in the graphs. The fluorescence intensities at the rupture sites were measured and normalized to the initial intensities (means ± SEM; *n* =20 cells from two independent experiments; ***, $P < 0.001$ from LA-FL by a mixed effect model). mEmerald-fused LA-FL, LAΔ26, LAΔ26-F627X, and PG are reproduction of Fig. 5D and E. **(B** and **E)** Magnified views of the indicated areas by orange boxes are shown (the second to fifth columns). A 2-μm diameter spot was laser-microirradiated to induce NE rupture (yellow arrowheads). Bars: 5 μm (the first column) and 2 μm (the second to fifth columns).

Source data for Fig. 2

A

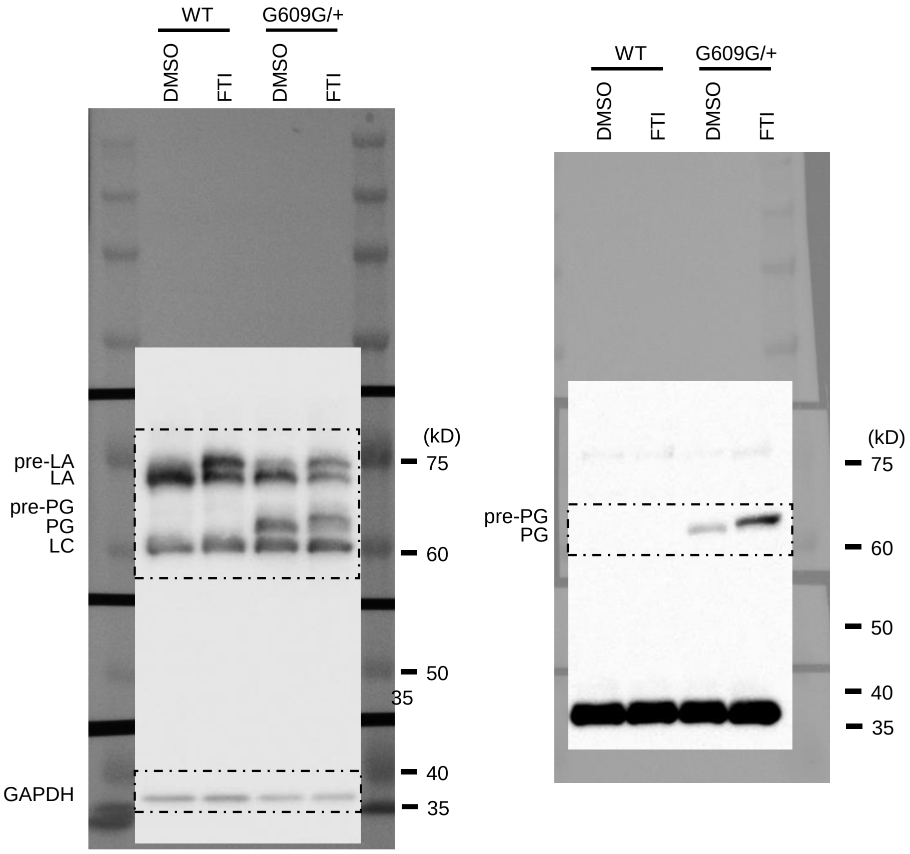

Fig. S9. Full length blots of Fig. S2A. Black dotted lines show the cropping areas. Brightness and contrast were not changed during the processing of these blots.

Source data for Fig. 4

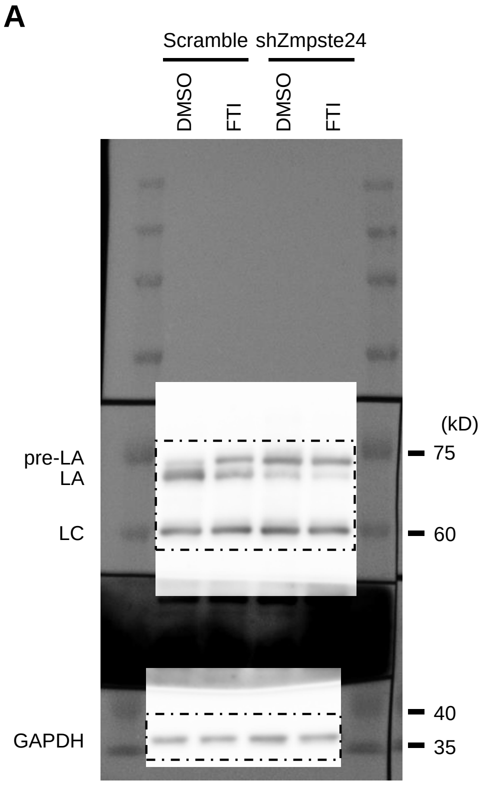

Fig. S10. Full length blots of Fig. 4A. Black dotted lines show the cropping areas. Brightness and contrast were not changed during the processing of these blots.

Source data for Fig. S1

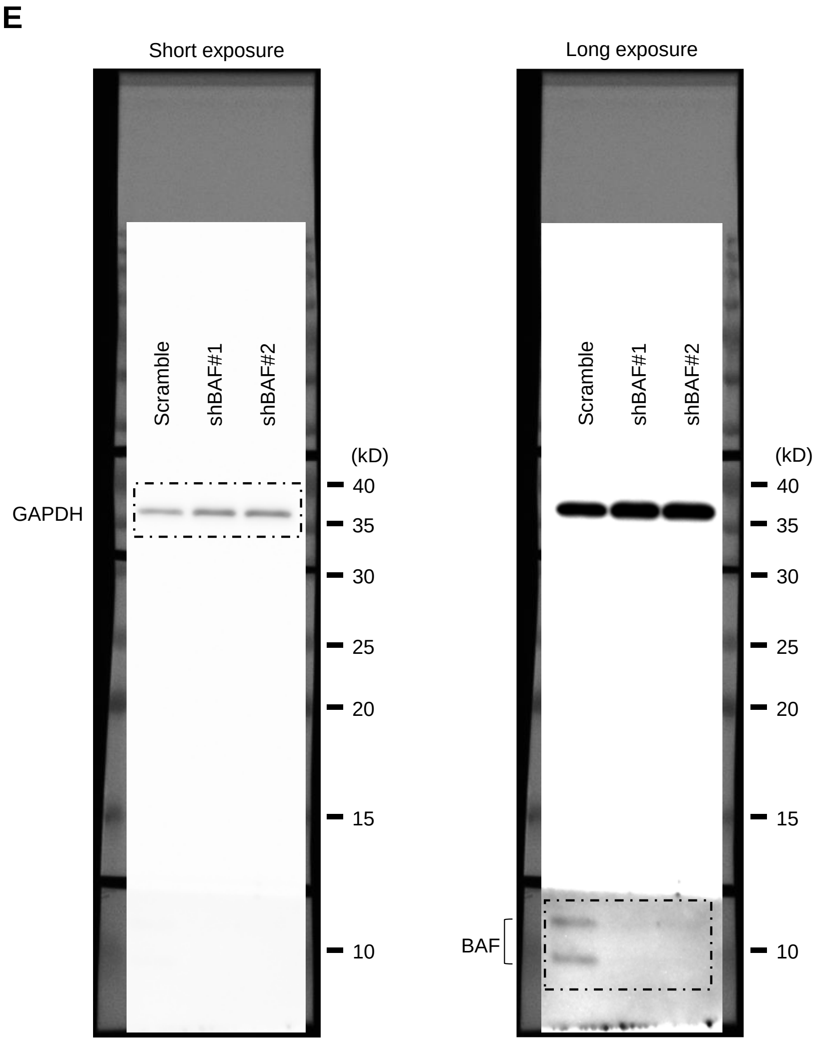

Fig. S11. Full length blots of Fig. S1E. Black dotted lines show the cropping areas. Brightness and contrast were not changed during the processing of these blots.

Source data for Fig. S3

B

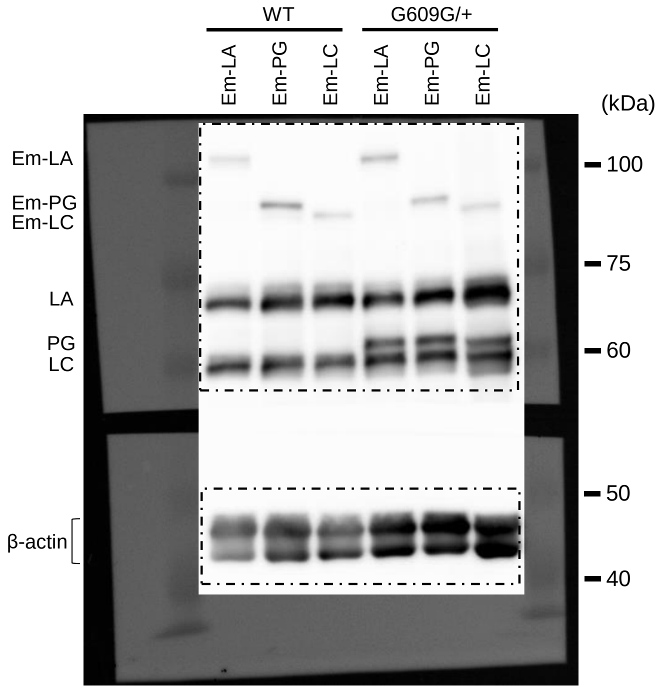

Fig. S12. Full length blots of Fig. S3B. Black dotted lines show the cropping areas. Brightness and contrast were not changed during the processing of these blots.

Source data for Fig. S5

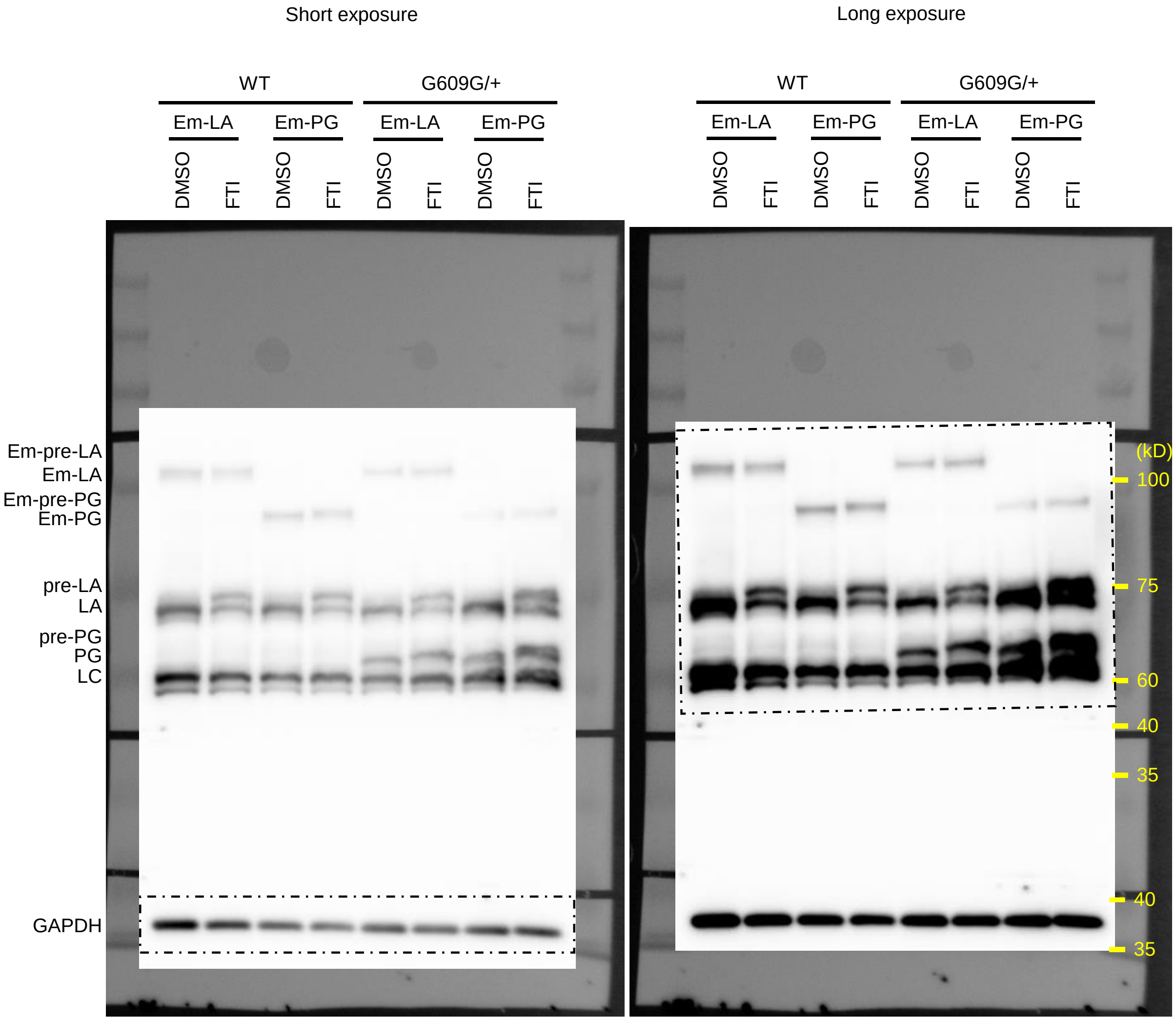

Fig. S13. Full length blots of Fig. S5. Black dotted lines show the cropping areas. Brightness and contrast were not changed during the processing of these blots.

Source data for Fig. S6

A

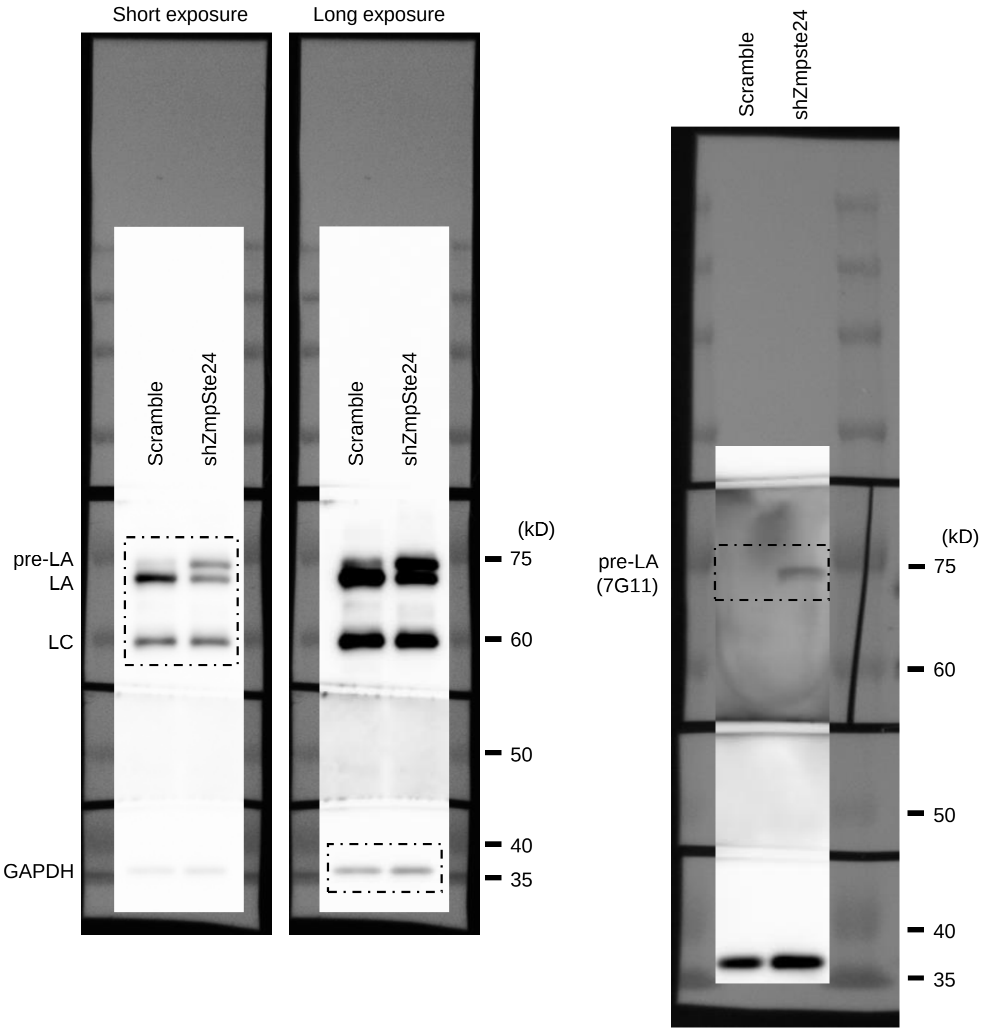

Fig. S14. Full length blots of Fig. S6. Black dotted lines show the cropping areas. Brightness and contrast were not changed during the processing of these blots.

Movie S1 (separate file). Movie of Fig. 1 C, (WT). Accumulation of cGAS-sfGFP at the rupture site and reentry of NLS-sfCherry into the nucleus. A WT cell expressing NLS-sfCherry and $cGAS^{E211A/D213A}$-sfGFP after the bead compression-induced NE rupture and imaged using Eclipse Ti2. Frames were collected every 30 s and displayed at 0.5 frame/s. Bar, 10 μm.

Movie S2 (separate file). Movie of Fig. 1 C, (G609G/+). Accumulation of cGAS-sfGFP at the rupture site and reentry of NLS-sfCherry into the nucleus. A G609G/+ cell expressing NLS-sfCherry and $cGAS^{E211A/D213A}$-sfGFP after the bead compression-induced NE rupture and imaged using Eclipse Ti2. Frames were collected every 30 s and displayed at 0.5 frame/s. Bar, 10 μm.

SI References

1. Y. Kono *et al.*, Nucleoplasmic lamin C rapidly accumulates at sites of nuclear envelope rupture with BAF and cGAS. *J Cell Biol* **221** (2022).
2. J. W. Littlefield, Three Degrees of Guanylic Acid--Inosinic Acid Pyrophosphorylase Deficiency in Mouse Fibroblasts. *Nature* **203**, 1142-1144 (1964).
3. M. Koi, H. Morita, M. Shimizu, M. Oshimura, Construction of mouse A9 clones containing a single human chromosome (X/autosome translocation) via micro-cell fusion. *Jpn J Cancer Res* **80**, 122-125 (1989).
4. Y. Goto, H. Kimura, Inactive X chromosome-specific histone H3 modifications and CpG hypomethylation flank a chromatin boundary between an X-inactivated and an escape gene. *Nucleic Acids Res* **37**, 7416-7428 (2009).
5. L. Sun, J. Wu, F. Du, X. Chen, Z. J. Chen, Cyclic GMP-AMP synthase is a cytosolic DNA sensor that activates the type I interferon pathway. *Science* **339**, 786-791 (2013).
6. R. M. Sears, K. J. Roux, Mechanisms of A-Type Lamin Targeting to Nuclear Ruptures Are Disrupted in LMNA- and BANF1-Associated Progerias. *Cells* **11** (2022).
7. F. A. Ran *et al.*, Genome engineering using the CRISPR-Cas9 system. *Nat Protoc* **8**, 2281-2308 (2013).
8. V. T. Chu *et al.*, Increasing the efficiency of homology-directed repair for CRISPR-Cas9-induced precise gene editing in mammalian cells. *Nat Biotechnol* **33**, 543-548 (2015).
9. Y. Guo, Y. Kim, T. Shimi, R. D. Goldman, Y. Zheng, Concentration-dependent lamin assembly and its roles in the localization of other nuclear proteins. *Mol Biol Cell* **25**, 1287-1297 (2014).
10. R. J. Platt *et al.*, CRISPR-Cas9 knockin mice for genome editing and cancer modeling. *Cell* **159**, 440-455 (2014).
